# Sports genomics research in Turkish athlete populations 2019 to 2026: a systematic review

**DOI:** 10.3389/fgene.2026.1921682

**Published:** 2026-10-23

**Authors:** Murat Kasap, Erkut Tutkun

**Affiliations:** 1 Faculty of Sport Sciences, Bartın University, Bartın, Türkiye; 2 Faculty of Sports Sciences, Bursa Uludağ University, Bursa, Türkiye

**Keywords:** athletes, athletic performance, polymorphism (genetics), sports genomics, systematic review, Turkey

## Abstract

**Background:**

Sports-genomics research in Turkish athlete populations has examined diverse sports, genetic approaches, and sport-related phenotypes. This systematic review mapped the scope, study populations, and methodological characteristics of this literature.

**Methods:**

This systematic review followed PRISMA 2020. Web of Science Core Collection, Scopus, PubMed/MEDLINE, SPORTDiscus with Full Text, Google Scholar, TRDizin, and DergiPark were searched for records published from 1 January 2019 to 1 April 2026. Original human studies were eligible when they included an identifiable Turkish athlete population and investigated inherited genetic or genomic variation in relation to a sport-related phenotype. Eligibility was determined by the characteristics of the study population and the availability of separately extractable data for Turkish athletes. Records were deduplicated and independently screened. Reports from confirmed shared cohorts were retained when they contributed non-overlapping eligible information and were linked during synthesis.

**Results:**

The database search yielded 1,740 records. After removal of 717 duplicates, 1,023 records underwent title and abstract screening and 396 full texts were assessed. Sixty-one database-derived reports and nine reports identified through backward reference-list checking and forward citation searching were included, yielding 70 reports from 58 operationally unique study populations. These populations comprised 3,201 athletes and 3,683 non-athlete controls. Eighteen reports originated from six confirmed shared-cohort clusters.

**Conclusion:**

This review provides a transparent evidence base for a cautious descriptive synthesis of Turkish sports-genomics research. The evidence is reported as a structured account of the study populations, genetic approaches, and phenotypic themes represented in the literature.

**Systematic Review Registration:**

https://doi.org/10.17605/OSF.IO/739CG.

## 1 Introduction

Athletic performance is a complex phenotype shaped by training, environmental exposures, nutrition, psychological factors, injury history, and inherited variation. Twin and family studies have supported a heritable contribution to several performance-related traits, while the magnitude and practical meaning of any individual genetic association remain context dependent (De Moor et al., 2007; Bray et al., 2009). Candidate variants in genes such as ACTN3, ACE, PPARA, and COL5A1 have frequently been examined in sport populations; however, many reported associations have not been consistently replicated across populations or sporting contexts (Ahmetov et al., 2016; Semenova et al., 2023).

Sports genomics is therefore moving from isolated candidate-variant studies towards approaches that can better reflect polygenic architecture, including genome-wide association studies (GWAS), exome-wide analyses, and aggregated genomic profiles. These approaches are not a simple replacement for candidate-gene research: they require adequate sample size, phenotype harmonisation, ancestry-aware analysis, appropriate multiple-testing control, independent replication, and transparent reporting (Rami et al., 2026; Bouchard, 2011).

Sports-genomics research involving Turkish athletes has examined candidate variants, genome-scale approaches, and diverse sport-related phenotypes. Evidence synthesis in this field requires a population-based definition of relevance. Turkish authorship or institutional affiliation does not establish that a study concerns Turkish athletes; eligibility must instead be determined from the athletes recruited and the extractability of the relevant data.

This systematic review examined original human sports-genomics research involving Turkish athlete populations published between 1 January 2019 and 1 April 2026. Its objectives were to identify eligible reports through a reproducible multi-database search, characterise the study populations, sports, genetic approaches, and phenotypic themes, and distinguish multiple reports from the same cohort. The review was intended as an evidence map and critical descriptive synthesis, not as a meta-analysis of individual variant effects.

## 2 Materials and methods

### 2.1 Design, reporting standard, and protocol

The review was designed and reported in accordance with the Preferred Reporting Items for Systematic Reviews and Meta-Analyses (PRISMA) 2020 statement (Page et al., 2021). A meta-analysis was not undertaken because of substantial heterogeneity in study populations, sports, phenotypes, genetic exposures, comparison groups, and analytical approaches. The protocol was registered prospectively on the Open Science Framework (OSF; https://doi.org/10.17605/OSF.IO/739CG).

### 2.2 Eligibility criteria

Studies were considered eligible if they met all of the following criteria: (1) original human research published from 1 January 2019 to 1 April 2026; (2) inclusion of athletes from a Turkish population, defined as athletes recruited in Türkiye, athletes representing Turkish national or club-level sport populations, or Turkish athlete participants forming an identifiable subgroup within a multinational study for whom relevant genetic data could be extracted separately; and (3) investigation of germline genetic variation in relation to sport participation, athletic performance or performance-related phenotypes, physiological or metabolic characteristics relevant to sport, injury susceptibility, or sport-related psychological traits. Eligible approaches included candidate-gene association studies and genome-wide or exome-wide approaches examining single-nucleotide variants, insertion/deletion polymorphisms, other inherited variants, or aggregated genomic profiles.

Studies were excluded if they did not include an identifiable Turkish athlete population or did not permit separate extraction for Turkish athletes; were reviews, systematic reviews, meta-analyses, editorials, commentaries, conference abstracts without sufficient primary data, case reports, animal studies, or *in vitro*/cell-culture studies; did not contain original genotype or genomic data relevant to an athletic or sport-related phenotype; or duplicated the same study population without providing additional eligible genetic or phenotypic information. When multiple reports from the same cohort supplied non-overlapping eligible information, they were retained as separate reports and linked to the same study population for synthesis.

Eligibility was determined by the characteristics of the study population and the availability of separately extractable data for Turkish athletes. Absence of reported ethics approval was not an automatic reason for exclusion because non-reporting did not establish that approval had not been obtained. No language restriction was applied at eligibility assessment. The operational eligibility framework is presented in Table 1.

**TABLE 1:** Operational eligibility framework for the systematic review.

Domain	Operational definition
Population	Turkish athletes recruited in Türkiye; Turkish national or club-level athlete populations; or separately extractable Turkish athlete subgroups in multinational studies
Exposure/concept	Inherited genetic or genomic variation, including single-nucleotide variants, insertion/deletion polymorphisms, other germline variants, GWAS/exome-wide markers, or aggregated genomic profiles
Comparator	Non-athlete controls, different athlete groups, genotype groups, or no comparator where appropriate to the design
Outcomes	Athlete status/participation, performance or performance-related phenotypes, physiological/metabolic traits, injury susceptibility, or sport-related psychological traits
Study designs	Original human cross-sectional, case-control, cohort, candidate-gene, genome-wide, and exome-wide research

### 2.3 Information sources and search strategy

The search covered Web of Science Core Collection, Scopus, PubMed/MEDLINE, SPORTDiscus with Full Text *via* EBSCOhost, Google Scholar, TRDizin, and DergiPark. Searches were limited to publications dated from 1 January 2019 through 1 April 2026.

Google Scholar was used as a supplementary source alongside bibliographic database. Because its search interface, ranking, and reported result counts are dynamic and not fully reproducible, the search date and screening limit were documented (Bramer, 2016). Consistent with a published recommendation to focus title screening on the first 200–300 Google Scholar results, the first 200 results returned by the systematic search were captured and screened (Haddaway et al., 2015). Google Scholar was not used as the sole basis for study identification.

In DergiPark and TRDizin, where a single long Boolean string was not reliably supported, smaller combinations derived from the same three concept blocks were used. Embase was not searched because institutional access was unavailable; this was considered a limitation of the search rather than an indication that Embase was not relevant.

The full database-specific search strings, search dates, filters, and a description of the TRDizin and DergiPark implementation are provided in Supplementary Table S2. Backward reference-list checking and forward citation searching identified nine additional eligible reports; these reports were shown separately in the PRISMA flow rather than added to the database-identification total. The database-specific search approaches and records identified before deduplication are summarised in Table 2.

**TABLE 2:** Databases, search strategies, and records identified before deduplication.

Source	Search concept/implementation	Records (n)
Web of science core collection	TS=((“sport” OR “athlete” OR “athletic performance” OR “spor”) AND (“genetic” OR “genetik”) AND (“polymorphism” OR “genotype” OR “polimorfizm”)); date audit to 1 April 2026	324
Scopus	TITLE-ABS-KEY((“sport” OR “athlete” OR “athletic performance” OR “spor”) AND (“genetic” OR “genetik”) AND (“polymorphism” OR “genotype” OR “polimorfizm”)); date audit to 1 April 2026	882
PubMed/MEDLINE	(“sport”[Title/Abstract] OR “athlete”[Title/Abstract] OR “athletic performance”[Title/Abstract] OR “spor”[Title/Abstract]) AND (“genetic”[Title/Abstract] OR “genetik”[Title/Abstract]) AND (“polymorphism”[Title/Abstract] OR “genotype”[Title/Abstract] OR “polimorfizm”[Title/Abstract]); 2019/01/01–2026/04/01	225
SPORTDiscus with full text	(TI (“sport” OR “athlete” OR “athletic performance” OR “spor”) OR AB (“sport” OR “athlete” OR “athletic performance” OR “spor”) OR SU (“sport” OR “athlete” OR “athletic performance” OR “spor”)) AND (TI (“genetic” OR “genetik”) OR AB (“genetic” OR “genetik”) OR SU (“genetic” OR “genetik”)) AND (TI (“polymorphism” OR “genotype” OR “polimorfizm”) OR AB (“polymorphism” OR “genotype” OR “polimorfizm”) OR SU (“polymorphism” OR “genotype” OR “polimorfizm”)); 01/2019–04/2026; AI search off; no full text or peer reviewed limit	84
Google scholar	(“Sport” OR “athlete” OR “athletic performance” OR “spor”) AND (“genetic” OR “genetik”) AND (“polymorphism” OR “genotype” OR “polimorfizm”); first 200 results systematically captured	200
TRDizin	Smaller combinations, primarily in abstract fields: Sport block (“spor”, “sport”, “athlete”, “athletic performance”); genetics block (“genetik”, “genetic”); variation block (“polimorfizm”, “polymorphism”, “genotype”). Date and duplicate checked	5
DergiPark	Twelve simpler searches combined: Sport/population block (“sport”, “spor”, “athlete”, “athletic performance”); genetics block (“genetic”, “genetik”); variation block (“polymorphism”, “polimorfizm”, “genotype”). Sixty-seven record views yielded 21 unique records; one post-cutoff record was removed	20
Total	Identification pool before within- and between-source deduplication	1,740

The search terms and combinations are listed in the table exactly as they were used during the search (in both Turkish and English).

### 2.4 Study selection and management of duplicate records

Exported records were combined in a master library. Within-source and between-source duplicates were removed using bibliographic identifiers where available and then by comparison of title, authorship, year, journal, and study details. The 1,740 database records yielded 1,023 unique records after removal of 717 duplicates. Two reviewers independently screened titles and abstracts against the prespecified eligibility criteria and retrieved the full texts of potentially eligible reports. Full-text reports were assessed independently against the same criteria. Disagreements were resolved through discussion and consensus.

At the report level, 627 records were excluded during title and abstract screening. The full texts of 396 database-derived reports were assessed, and none were not retrieved. Of these, 335 reports were excluded at full-text assessment and 61 reports were included. Nine eligible reports identified through backward reference-list checking and forward citation searching were included, yielding a total of 70 included reports.

### 2.5 Data extraction and cohort linkage

A standardised extraction form was used to record bibliographic information; database source(s); study design; country and athlete population; sport; athlete and comparator sample sizes; genetic approach and variants; phenotype; primary finding; and information relevant to cohort linkage. The extraction form also recorded whether ethics approval, Hardy-Weinberg equilibrium assessment, power/sample-size justification, adjustment for confounding, multiple-testing procedures, and replication were reported. These fields were coded as reported, not reported, unclear, or not applicable as appropriate; absence of reporting was not interpreted as proof that the underlying procedure had not occurred.

Potentially overlapping samples were evaluated using author overlap, recruitment setting, sport, recruitment period where available, sample size, demographic characteristics, control source, and the genetic and phenotypic information reported. Reports were considered to arise from a confirmed shared cohort only when the available evidence supported this conclusion. Participants were counted once within each operationally unique population in aggregate population totals. Six confirmed shared-cohort clusters encompassed 18 reports, and the final dataset included 58 operationally unique study populations.

### 2.6 Synthesis strategy

A meta-analysis of individual variants was not undertaken because the evidence base was heterogeneous in sport, athlete definition, phenotype, comparison group, genetic model, and analytic approach. Results were presented as a structured descriptive synthesis. Reports were grouped by sport, genetic approach, phenotypic theme, and study-population linkage. Candidate-gene findings were not treated as established associations solely because statistical significance was reported in a single study. In interpreting the evidence, we considered sample size, phenotype definition, ancestry and population structure, multiple testing, control selection, replication, and the risk of selective reporting.

Descriptive proportions for genetic approach and primary phenotypic theme used the 70 included reports as their denominator. Participant totals were calculated across the 58 operationally unique study populations after confirmed shared-cohort merging and were not interpreted as a simple sum of participants across all published reports.

### 2.7 Certainty, publication bias, and limitations of the search

Formal tests for small-study effects or publication bias were not performed because the review did not pool a sufficiently homogeneous set of effect estimates. The potential for publication bias and selective-reporting bias was considered qualitatively in the interpretation of the evidence. The search included both international and Turkish bibliographic sources and did not apply a language restriction at eligibility assessment. Nonetheless, Embase could not be searched because institutional access was unavailable, and Google Scholar results were dynamic; these factors may have limited retrieval completeness.

### 2.8 Ethics and data availability

Ethics approval was not required for this systematic review because it synthesised published and publicly available information. The study-selection data, report-level extraction table, cohort-linkage table, PRISMA flow data, and full-text exclusion log are available in the accompanying supplementary materials.

### 2.9 Supplementary materials

## 3 Results

### 3.1 Study selection

The database search identified 1,740 records. After removal of 717 within-source and between-source duplicates, 1,023 unique records underwent title and abstract screening. A total of 627 records were excluded at this stage, leaving 396 reports for full-text assessment. All 396 full texts were retrieved. Of these, 335 reports did not meet the eligibility criteria, whereas 61 database-derived reports were included. Nine additional eligible reports identified through backward reference-list checking and forward citation searching were included, resulting in 70 included reports. The PRISMA flow is summarised in Table 3 and Figure 1.

**TABLE 3:** PRISMA 2020 study-selection counts.

Stage	Measure	n
Identification	Records identified from databases	1,740
Identification	Reports identified through backward reference-list checking and forward citation searching	9
Deduplication	Duplicates removed from database records	717
Screening	Unique records screened by title and abstract	1,023
Screening	Records excluded by title and abstract	627
Eligibility	Full-text reports assessed for eligibility	396
Eligibility	Full-text reports not retrieved	0
Eligibility	Full-text reports excluded	335
Included	Database-derived reports included	61
Included	Reports included from backward reference-list checking and forward citation searching	9
Included	Total included reports	70
Included	Operationally unique study populations after confirmed cohort merging	58

The corresponding PRISMA 2020 flow diagram is presented as Figure 1; its numerical content is identical to Table 3.

**TABLE 4:** Supplementary files supporting transparency and reproducibility.

File	Content
Supplementary Table S1	Report-level characteristics and main findings for all 70 included reports
Supplementary Table S2	Full database-specific search strategies, limits, and search dates
Supplementary Table S3	Full-text reports assessed for eligibility and explicit reason for exclusion
Supplementary Table S4	Cohort-linkage assessment and population-level participant totals
Supplementary Data S1	Title/abstract screening and duplicate-management audit trail

PRISMA study-selection counts and the supplementary materials supporting transparency and reproducibility are summarised in Table 3 and 4, respectively.

**FIGURE 1:**
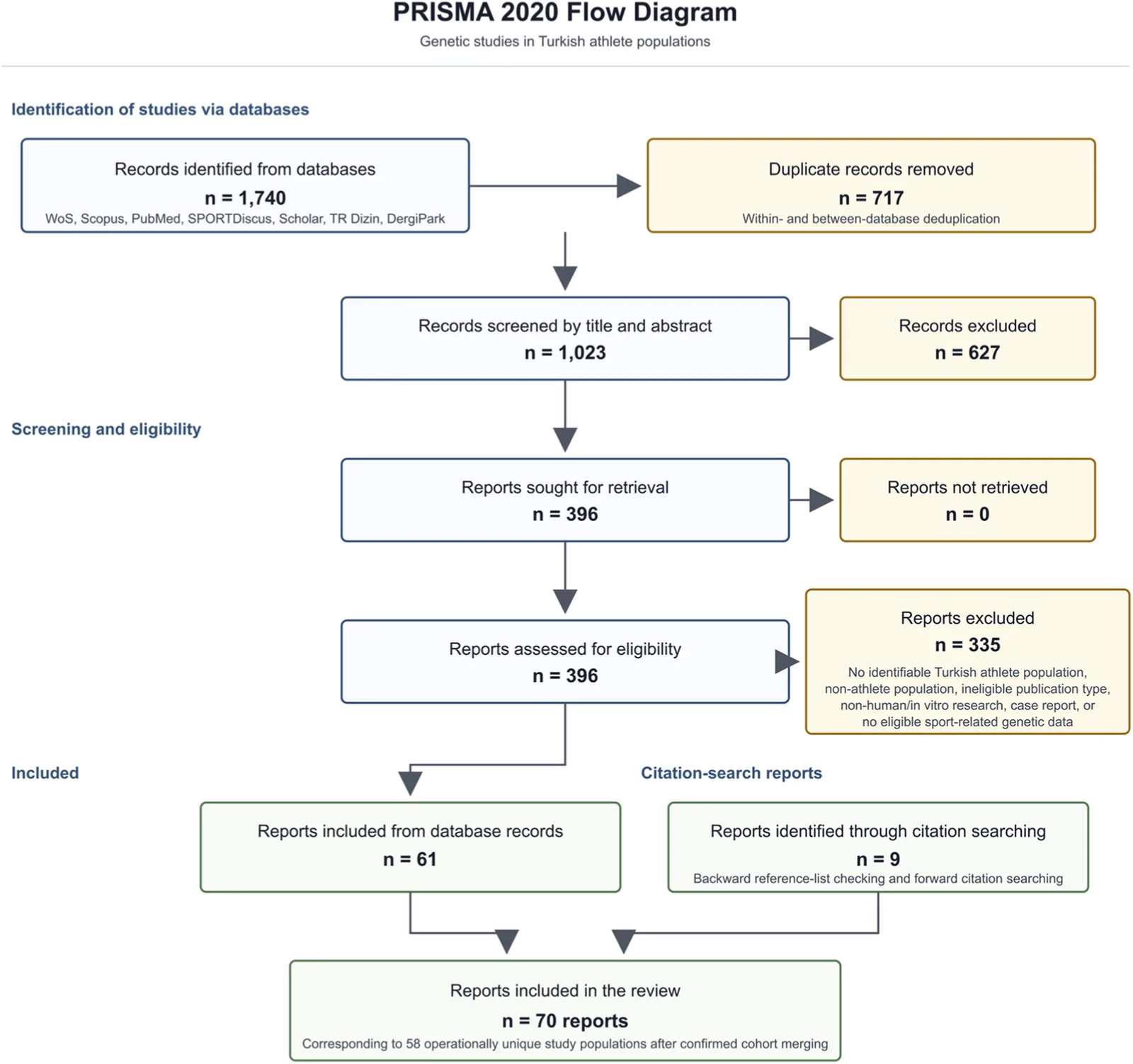
PRISMA 2020 flow diagram of study identification, screening, eligibility assessment, and inclusion.

### 3.2 Characteristics of included reports and study populations

The 70 included reports represented 58 operationally unique study populations. After confirmed shared-cohort merging, these populations comprised 3,201 athletes and 3,683 non-athlete controls (total, 6,884 participants). Six confirmed shared-cohort clusters encompassed 18 reports (25.7%). Participant totals were calculated at the study-population level. Where reports were confirmed to originate from the same cohort, participants were counted once only. Therefore, the totals did not equal the simple sum of sample sizes reported across all 70 articles. Key characteristics of the included reports and study populations are summarised in Table 5. A complete report-level description of all included studies is provided in Supplementary Table S1. (Akkoç et al., 2020; Aslan, 2019; Aslan et al., 2024; Bayraktar et al., 2023; Bilen, 2021; Bilen and Elioz, 2023; BOZYURT and YAMAK, 2024; Bulğa et al., 2025; Bulğay et al., 2021; Bulgay et al., 2021a; Bulgay et al., 2021b; Bulğay et al., 2022; Bulğay et al., 2023; Bulgay et al., 2023a; Bulgay et al., 2023b; Bulgay et al., 2023c; Bulgay et al., 2024; Bulgay et al., 2025; Canikli et al., 2022; Cerit et al., 2025; Chauchat et al., 2024; Çığırtaş et al., 2024; Demirci et al., 2023; Dogan et al., 2022; Doğan et al., 2022; Duran and Eliöz, 2024; Eken et al., 2019; Eken et al., 2021a; Eken et al., 2021b; Eken et al., 2021c; Eken et al., 2023; Ercan and Zulfinaz, 2020; Erkan et al., 2022; Gözler et al., 2021; Horozoglu et al., 2022; Kanlı and Çolak, 2024; Kaynar et al., 2019; Kaynar et al., 2021; Kaynar et al., 2025; Kazan et al., 2024; Kazan et al., 2025; Kazancı et al., 2023; Koku et al., 2019; Kozuma et al., 2025; Kurtulu et al., 2023; Kurtuluş et al., 2019; Kurtuluş et al., 2025; Muhan et al., 2023; Nursal et al., 2021; Nursal et al., 2023; Pekdaş et al., 2024; Piri et al., 2025; Pirim et al., 2024; Polat et al., 2020; Se et al., 2025; Sökmen et al., 2026; Söyler et al., 2024; Tacal Aslan et al., 2021; Tunalı et al., 2019; Ulucan et al., 2020; Unve et al., 2024; Unver et al., 2022; Unver et al., 2024; Usaç et al., 2020; Yamak et al., 2021; Yiğit et al., 2020; Yıldız et al., 2025; Yılmaz et al., 2024; Zileli et al., 2023a; Zileli et al., 2023b).

**TABLE 5:** Characteristics of the included reports and study populations.

Characteristic	n (%)	Notes
Review scope	​	​
Included reports	70	​
Operationally unique study populations	58	​
Athlete participants across operationally unique study populations	3,201	​
Non-athlete controls across operationally unique study populations	3,683	​
Total participants across operationally unique study populations	6,884	​
Reports from confirmed shared cohorts	18 (25.7%)	Six confirmed shared-cohort clusters; participants were counted once in population totals
Genetic approach	​	​
Candidate-gene association study	64 (91.4%)	​
Genome-wide association study (GWAS)	3 (4.3%)	​
Exome-wide approach	1 (1.4%)	​
Candidate-gene analysis using exome data	1 (1.4%)	​
Targeted analysis of an inherited insertion/deletion (indel) variant	1 (1.4%)	​
Primary phenotypic theme	​	​
Athletic performance or performance-related phenotype	28 (40.0%)	​
Athlete status/participation or genotype distribution	28 (40.0%)	​
Sport-related psychological trait	5 (7.1%)	​
Injury susceptibility	5 (7.1%)	​
Physiological, metabolic, or anthropometric trait	4 (5.7%)	​

Percentages for genetic approach and primary phenotypic theme were calculated using the 70 included reports as the denominator. Each report was assigned one primary genetic approach and one primary phenotypic theme for descriptive counting. Participant totals represent unique individuals after confirmed shared cohorts were merged: 3,201 athletes and 3,683 non-athlete controls across 58 operationally unique study populations. These values should not be interpreted as the sum of participant numbers reported in all articles because some participants contributed data to more than one report. Six confirmed shared-cohort clusters encompassed 18 reports. GWAS, genome-wide association study; indel, insertion/deletion variant.

### 3.3 Genetic approaches

Candidate-gene association studies accounted for 64 of the 70 reports (91.4%). Three reports (4.3%) used GWAS designs, one report (1.4%) used an exome-wide approach, one (1.4%) used exome data for a candidate-gene analysis, and one (1.4%) evaluated a targeted inherited indel variant. Genome-scale approaches were represented, but candidate-gene designs remained the predominant approach. These categories were assigned at the report level; reports from the same study population could therefore contribute different genetic approaches when they addressed non-overlapping analyses.

### 3.4 Phenotypic themes

The most frequent primary themes were athletic performance or a performance-related phenotype and athlete status, sport participation, or genotype distribution (28 reports each, 40.0%). Five reports (7.1%) addressed sport-related psychological traits, five (7.1%) injury susceptibility, and four (5.7%) physiological, metabolic, or anthropometric traits. Each report was assigned one primary theme for descriptive counting; studies reporting more than one outcome were not counted repeatedly across themes. The distribution is displayed in Figure 2.

**FIGURE 2:**
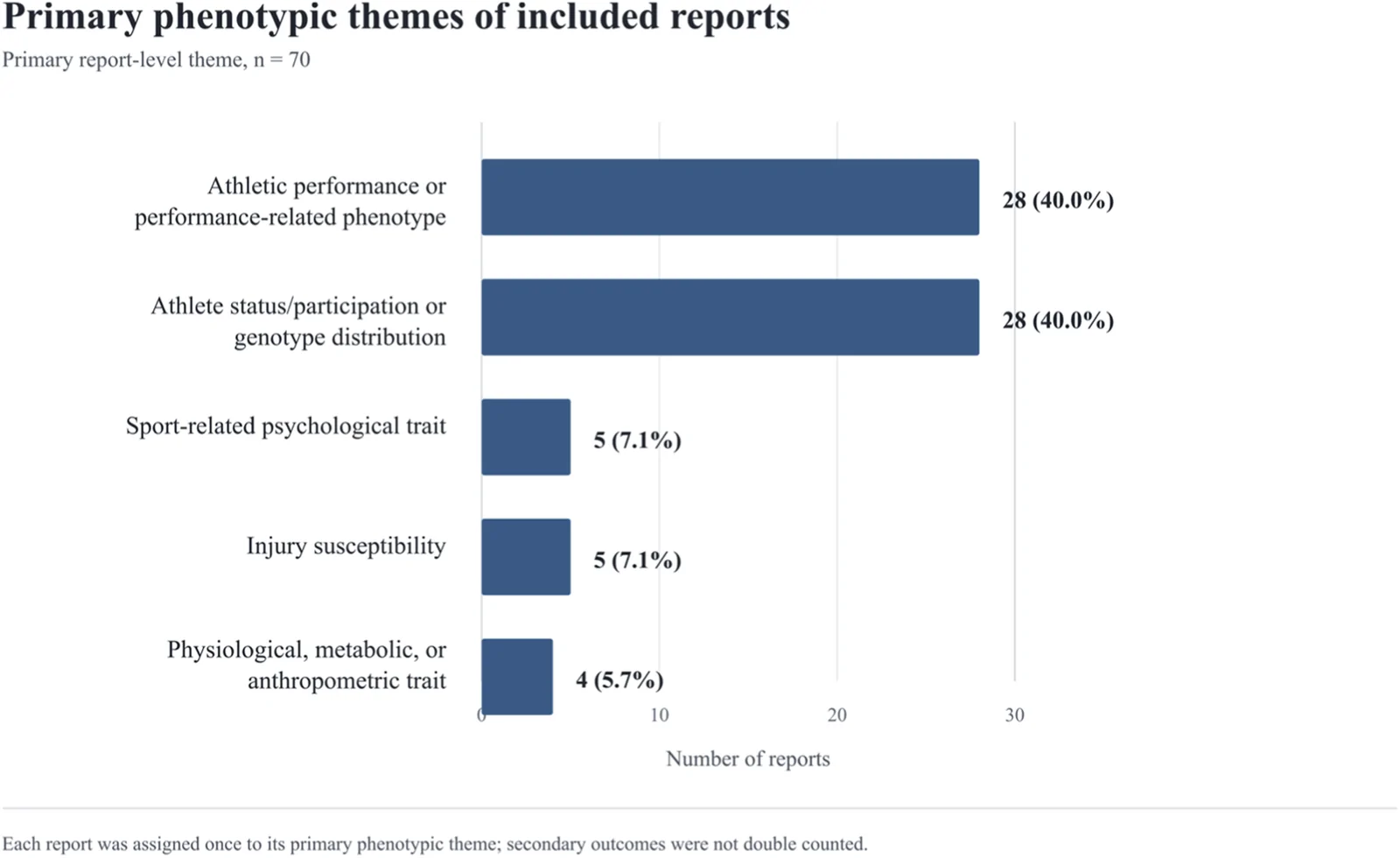
Primary phenotypic themes of included reports. Each report was assigned once to its primary phenotypic theme; secondary outcomes were not double counted.

### 3.5 Sports represented in the study populations

Football was the most frequently represented single-sport category, comprising 14 operationally unique populations and 652 athletes. Nine populations (1,256 athletes) were classified as multi-sport or not reliably separable by sport because reports described mixed athlete samples without extractable sport-specific participant distributions. Volleyball, badminton, wrestling, and cross-country skiing were also represented by multiple populations. Table 6 presents the population-level distribution; the complete population linkage and totals are provided in Supplementary Table S4.

**TABLE 6:** Sports and participant counts across operationally unique study populations.

Sport/category	Unique populations	Athletes	Controls	Total
Multi-sport/not separable	9	1,256	953	2,209
Football	14	652	641	1,293
Volleyball	5	135	557	692
Badminton	1	90	557	647
Wrestling only	2	113	195	308
Cross-country skiing	3	83	130	213
Endurance sports	1	60	110	170
Running	3	113	50	163
Arm wrestling	2	92	68	160
Basketball	2	45	72	117
Kickboxing	1	12	101	113
Bodybuilding	3	61	50	111
Badminton and wrestling mixed-sport cohort	1	107	0	107
Triathlon	1	47	46	93
Anaerobic sports	1	43	43	86
Athletics	1	60	20	80
Ultimate frisbee	1	30	30	60
Basketball and volleyball mixed-sport cohort	1	53	0	53
Rugby	1	22	30	52
Futsal/football	1	48	0	48
Combat sports	1	40	0	40
HADO	1	8	30	38
Ice hockey	1	21	0	21
Ironman	1	10	0	10
Total	58	3,201	3,683	6,884

Counts were calculated at the operationally unique population level. Combined-sport categories represent distinct study populations in which athletes were recruited from more than one sport. These cohorts were counted once only under the combined category and were not additionally included in the corresponding single-sport rows. Accordingly, the 107 athletes in the “Badminton and wrestling” cohort were separate from the 113 athletes reported in the wrestling-only populations; likewise, the “Basketball/volleyball” cohort was separate from the basketball-only and volleyball-only populations. Participant counts therefore should not be summed across overlapping sport labels.

### 3.6 Cohort linkage and interpretive boundaries

Six shared-cohort clusters were identified. These clusters encompassed a total of 18 reports that drew wholly or partly on the same underlying athlete and/or control populations. Reports were retained separately only when they provided non-overlapping eligible genetic or phenotypic information. This linkage avoided double counting in the population-level totals while preserving report-level evidence in the descriptive synthesis.

## 4 Discussion

### 4.1 Principal findings

This systematic review identified 70 eligible reports representing 58 operationally unique study populations. The evidence covered a broad but heterogeneous literature on genetic variation in relation to athlete status, sport participation, athletic performance and performance-related phenotypes, injury susceptibility, sport-related psychological traits, and physiological, metabolic, or anthropometric traits. Studies differed substantially in sport, participant characteristics, genetic approach, comparison group, and outcome definition. This heterogeneity did not support directly combinable estimates of genetic effects; therefore, the findings were interpreted as a qualitative evidence map of the scope, patterns, and methodological limitations of sports-genomics research in Turkish athlete populations.

Athletic performance and related phenotypes were the most frequent primary theme, followed closely by athlete status, sport participation, or genotype distribution. This pattern indicated that much of the literature focused on whether particular variants were distributed differently across athlete groups or were associated with a selected performance-related measure. Injury susceptibility, psychological traits, and physiological, metabolic, or anthropometric outcomes were less common but showed that the field extended beyond a narrow performance-only framing. The thematic categories were descriptive and report based; they did not establish that the evidence was equally mature or equally credible across themes.

### 4.2 Candidate-gene predominance and the emergence of genomic approaches

Candidate-gene association studies accounted for 64 of the 70 included reports. Three GWAS reports, one exome-wide report, and one report using exome data for a candidate-gene analysis were also identified. Together, these reports showed that more than one genetic research approach had been used in Turkish athlete populations. However, the limited number of genome-scale reports did not establish a sustained transition away from candidate-gene research. Candidate-gene designs remained the dominant approach.

Genome-wide and exome-wide strategies can be valuable because athletic performance is plausibly polygenic and because they can reduce reliance on a small set of repeatedly selected variants. However, they also introduce demanding requirements: sufficiently large and well-characterised samples, harmonised phenotype definitions, ancestry-aware analyses, appropriate control of multiple testing, replication in independent samples, and transparent reporting of quality control and analytic decisions. Consequently, the implication of the present review is not that GWAS, exome sequencing, or polygenic scores should replace all candidate-gene research. Rather, future studies should select an approach that is proportionate to the question and sample, and should prioritise collaborative designs that can support robust discovery and replication.

### 4.3 Diversity of sports, phenotypes, and study populations

The evidence base included football, volleyball, wrestling, cross-country skiing, running and athletics, combat sports, and several smaller or mixed-sport populations. Football was the most frequently represented single-sport category, whereas a sizeable proportion of participants arose from mixed-sport populations for which sport-specific allocation could not be reliably extracted. This diversity captured the breadth of Turkish sport settings, but it also limited direct comparison: a genotype associated with athlete status in one sport could not be assumed to have the same association with an endurance-related phenotype, an injury outcome, or a psychological trait in another sport.

The linkage of reports to 58 operationally unique populations was particularly important for interpretation. Six confirmed shared-cohort clusters encompassed 18 reports. Retaining non-overlapping reports from the same cohort preserved useful genetic or phenotypic information, but treating them as wholly independent would have exaggerated the breadth of the underlying participant base. Merging them at the population level avoided double counting while allowing report-level findings to remain visible. Future reports should make cohort provenance, recruitment sites, recruitment periods, participant overlap, and reuse of comparator samples clearer to facilitate evidence synthesis.

### 4.4 Methodological and reporting considerations

Several features central to the credibility of genetic association evidence required careful consideration when interpreting this literature. Small samples could reduce precision and statistical power, increase the chance of unstable effect estimates, and amplify winner’s-curse effects. Lack of independent replication constrained confidence in isolated positive associations. In addition, unaddressed population stratification, heterogeneous athlete definitions, variable phenotype measurement, selective genetic-model choice, and incomplete handling of multiple comparisons could each contribute to apparent inconsistency across studies. These considerations were especially relevant in a literature dominated by candidate-variant analyses, where numerous plausible tests may have been undertaken within a limited sample.

A missing statement about Hardy-Weinberg equilibrium, power calculation, covariate adjustment, or multiple-testing control was described as not reported or unclear, not as proof that the procedure was not performed. Hardy-Weinberg equilibrium was context dependent and was most informative when evaluated in an appropriate control population. Nevertheless, transparent reporting of these elements was essential because it enabled readers to assess analytical decisions and the plausibility of reported associations.

### 4.5 Interpretation of findings in the literature context

This review showed that sports-genomics research in Turkish athlete populations was dominated by candidate-gene association designs, while genome-wide and exome-based approaches were also represented. Heterogeneity in sports, participant characteristics, genetic methods, and phenotypic outcomes did not support synthesis into a single genetic-effect estimate. The evidence was therefore interpreted as a qualitative account of the scope and methodological diversity of the literature, rather than as a collection of definitive effects of individual variants on athlete status or performance.

The review had limitations. Embase could not be searched because institutional access was unavailable, and Google Scholar results were dynamic; both factors may have affected retrieval completeness. The included literature was heterogeneous, precluding a reliable meta-analysis of individual variant effects. Participant totals were adjusted for confirmed shared cohorts, but cohort overlap could not be excluded when reports did not provide enough information to establish or rule out shared recruitment. Finally, the review focused on Turkish athlete populations and did not provide a comparative assessment of national research systems; its implications therefore warranted cautious application outside this evidence base.

### 4.6 Implications for future research

Future Turkish sports-genomics research would benefit from prospectively defined phenotypes, transparent athlete and control selection, preregistered or clearly justified genetic models, adequate sample-size planning, and complete reporting of genotype quality control, effect estimates, uncertainty, and multiple-testing procedures. Multicentre collaboration and data harmonisation would make independent replication and ancestry-aware analyses more feasible. Studies using GWAS, exome-wide approaches, or polygenic scores should incorporate discovery and replication stages where possible and should avoid overstating the predictive value of genetic information. Such steps would improve reproducibility without implying that any single analytical platform is universally preferable.

## 5 Conclusion

This systematic review identified 70 eligible reports representing 58 operationally unique Turkish athlete study populations. The evidence base spanned multiple sports and sport-related phenotypes and included candidate-gene, genome-wide, and exome-wide approaches; however, candidate-gene studies remained predominant. The diversity of populations, phenotypes, comparison groups, and analytical approaches supported a structured descriptive synthesis rather than pooled genetic-effect estimates.

Population-based eligibility criteria, a systematic search of international and Turkish sources, transparent documentation of study selection, and linkage of reports from confirmed shared cohorts strengthened the interpretability of this evidence base. Individual association findings warranted cautious interpretation, particularly where samples were small, replication was unavailable, phenotype definitions differed, population structure was insufficiently addressed, or multiple-testing and quality-control procedures were unclear.

The field required transparent reporting, better-defined athlete and comparator populations, adequate sample-size planning, harmonised phenotypes, ancestry-aware analysis, appropriate control of multiple testing, and independent replication. Genome-wide, exome-wide, and aggregated genomic approaches could complement candidate-gene research when their design and sample requirements were met; they did not substitute for rigorous study design. These priorities supported the reproducibility and cumulative value of sports-genomics research involving Turkish athlete populations.

## Data Availability

The original contributions presented in the study are included in the article/Supplementary Material, further inquiries can be directed to the corresponding author.
