# Supplementary material for: Sports genomics research in Turkish athlete populations 2019 to 2026: a systematic review
Source: Front Genet. 2026 Oct 23;17:1921682. doi: 10.3389/fgene.2026.1921682 (PMC13739473; doi:10.3389/fgene.2026.1921682)
Supplement: Supplementary file 1 [file DataSheet1.pdf]

Supplementary Table S1 Detailed characteristics of included reports

Report-level data are shown for all 70 included reports. Reports sharing a Population ID may derive from the same confirmed cohort and were retained because they reported distinct genetic analyses or phenotypic information.

Report	Population and sample	Sport and approach	Genetic marker(s)	Primary phenotypic theme	Main finding
Y0956 (2019/12/01/ara2019) ELİT TÜRK GÜREŞÇİLERİNİN ANJİOTENSİN I-DÖNÜŞTÜRÜCÜ ENZİM GEN POLİMORFİZMİ VE VO_{2MAX} DÜZEYLERİ İLE ATLETİK PERFORMANS ARASINDAKİ İLİŞKİNİN BELİRLENMESİ	POP-03 Single/independent Athletes 46; controls 58; total 104	Wrestling Candidate-gene association study	ACE I/D	Athletic performance or performance-related phenotypes	No significant association was reported between ACE I/D and VO_2max or athletic performance.
Y0965 (2025; 2025/09/01/2025) ELİT ERKEK TÜRK SPORCULARINDA ACTN3 R577X POLİMORFİZMİNİN DEĞERLENDİRİLMESİ	POP-04 Confirmed shared cohort Athletes 158; controls 56; total 214	Multiple sports Candidate-gene association study	ACTN3 R577X	Athlete status/participation or genotype distribution	ACTN3 R577X genotype and allele frequencies did not differ significantly between athlete groups and controls or across sports.
Y0970 (2024/12/01/ara2024) Investigation of PPAR-Alpha gene polymorphism in female's soccer players.	POP-05 Single/independent Athletes 24; controls 24; total 48	Women's soccer Candidate-gene association study	PPARA rs4253778	Athlete status/participation or genotype distribution	No significant difference in PPARA rs4253778 genotype or allele distribution was reported between football players and sedentary women.
Y0981 (2023) Genetic differences in peroxisome proliferator-activated receptor alpha gene in endurance athletes (long distance runners) and power/endurance athletes (wrestlers, football players)	POP-04 Confirmed shared cohort Athletes 158; controls 56; total 214	Multiple sports Candidate-gene association study	PPARA rs4253778	Athlete status/participation or genotype distribution	PPARA intron 7 G/C genotype and allele frequencies did not differ significantly between athlete subgroups and non-athletes.
Y0984 (2024) HADO Sporcularında Alfa-Aktin-3 (ACTN3) rs1815739 Polimorfizminin Dağılımı	POP-06 Single/independent Athletes 8; controls 30; total 38	HADO Candidate-gene association study	ACTN3 rs1815739	Athlete status/participation or genotype distribution	No significant difference in ACTN3 rs1815739 distribution was reported between HADO athletes and controls.
Y0992 (2021) Farklı Branşlardaki Sporcuların Kollajen Tip V gen (COL5A1) rs12722 Polimorfizmlerinin Dağılımı	POP-07 Single/independent Athletes 115; controls 0; total 115	Multiple sports Candidate-gene association study	COL5A1 rs12722	Athlete status/participation or genotype distribution	No significant difference in COL5A1 rs12722 genotype or allele distribution was reported across sports.
Y1003 (2021) Türk Futbolcularda Alfa-Aktinin-3 (ACTN3) Ve Anjiyotensin Dönüştürücü Enzim (ACE) Polimorfizmleri Atletik Performans için Bir Biyobelirteç Olabilir mi?	POP-08 Single/independent Athletes 21; controls 105; total 126	Soccer Candidate-gene association study	ACTN3 ve ACE	Athletic performance or performance-related phenotypes	ACTN3 and ACE polymorphism distributions were compared between professional football players and controls; numerical results required confirmation from the full text.

Report	Population and sample	Sport and approach	Genetic marker(s)	Primary phenotypic theme	Main finding
Y1021 (2020)<br>Determination of SLC6A4 promoter L/S polymorphism in professional volleyball players and comparison of the literature; Profesyonel voleybolcularda Slc6a4 promotor L/S polimorfizminin belirlenmesi ve literatur karsilasti.	POP-09<br>Single/independent<br>Athletes 21; controls 0; total 21	Voleybol<br>Candidate-gene association study	SLC6A4 L/S	Athlete status/participation or genotype distribution	The LL genotype and L allele were reported more frequently in professional volleyball players; no non-athlete control group was included.
Y1026 (2021)<br>Mücadele sporcularında SLC6A4 geni 5-HTTLPR polimorfizmi ile saldırganlık düzeyleri arasındaki ilişkinin incelenmesi	POP-10<br>Confirmed shared cohort<br>Athletes 40; controls 0; total 40	Combat sports<br>Candidate-gene association study	SLC6A4 5-HTTLPR/rs25531	Sport-related psychological traits	Associations between 5-HTTLPR and rs25531 variants and aggression were reported in elite combat-sport athletes.
Y1029 (2020)<br>The evaluation of rs1805086 and rs1805065 polymorphisms in mstn gene and anthropometric properties of national and amateur arm wrestlers Evaluación de los polimorfismos rs1805086 y rs1805065 en el gen mstn y las propiedades antropométricas de los luchadores de brazo nacionales y amateurs	POP-11<br>Single/independent<br>Athletes 45; controls 34; total 79	Arm wrestling<br>Candidate-gene association study	MSTN rs1805086/rs1805065	Physiological, metabolic, or anthropometric traits	No significant difference in MSTN variant distribution was reported between national and amateur arm wrestlers; anthropometric measures differed by sex.
Y1032 (2021)<br>THE EXAMINATION OF THE EFFECTS OF PPAR-A (RS4253778) ON SERUM _x000D_ LIPIDS IN ELITE CROSS-COUNTRY SKIERS	POP-12<br>Confirmed shared cohort<br>Athletes 34; controls 0; total 34	Cross-country skiing<br>Candidate-gene association study	PPARA rs4253778	Physiological, metabolic, or anthropometric traits	No significant association was reported between PPARA rs4253778 genotypes and serum lipid levels; the GG genotype was most frequent (67.64%).
Y1037 (2019)<br>Kas dokusu fizyolojisinde rol oynayan iki aday gen varyantının alt ekstremite kas enduransı yönünden farklı mevkilerde oynayan futbolcularda incelenmesi	POP-13<br>Single/independent<br>Athletes 32; controls 0; total 32	Soccer<br>Candidate-gene association study	AMPD1 ve CKMM	Athletic performance or performance-related phenotypes	The AMPD1 CT genotype differed from CC for some performance and anthropometric indicators; all athletes were GG for CKMM.
Y1041 (2019)<br>Türk Kökenli Futbolcularda Apolipoprotein-E Genotiplerinin Belirlenmesi	POP-14<br>Single/independent<br>Athletes 22; controls 0; total 22	Soccer<br>Candidate-gene association study	APOE rs429358/rs7412	Athlete status/participation or genotype distribution	APOE ε3/ε3 was the most frequent genotype; ε2/ε2 and ε4/ε4 were not detected. Because no control comparison was available, the finding on athlete status was descriptive.
Y1064 (2019)<br>Determination of HIF1-Ars11549465 Polymorphism in Elite_x000D_ Skiers	POP-12<br>Confirmed shared cohort<br>Athletes 34; controls 0; total 34	Cross-country skiing<br>Candidate-gene association study	HIF1A rs11549465	Athlete status/participation or genotype distribution	In elite Turkish cross-country skiers, the CC genotype and C allele predominated for HIF1A rs11549465; the finding was descriptive because no control group was included.
Y1088 (2022)<br>Association between NOS3 intron 4 VNTR variant and sports performance	POP-15<br>Single/independent<br>Athletes 136; controls 150; total 286	Multiple sports<br>Candidate-gene association study	NOS3 intron 4 VNTR	Athlete status/participation or genotype distribution	The NOS3 4a/4a genotype was reported more frequently in Turkish athletes than sedentary controls; allele distribution did not differ significantly.

Report	Population and sample	Sport and approach	Genetic marker(s)	Primary phenotypic theme	Main finding
Y1089 (2021)<br>Preliminary Findings of the Distribution of Alpha-2-Adrenergic Receptor (ADRA2A) rs1800544 Polymorphisms in Kickboxing Players	POP-17<br>Single/independent<br>Athletes 12; controls 101; total 113	Kickboks<br>Candidate-gene association study	ADRA2A rs1800544	Athlete status/participation or genotype distribution	No significant difference in ADRA2A rs1800544 genotype or allele distribution was reported between Turkish-origin kickboxers and sedentary controls.
Y1092 (2020)<br>VEGFR2 Gene Polymorphism and Aerobic Performance in Turkish Elite Endurance Athletes	POP-18<br>Single/independent<br>Athletes 60; controls 110; total 170	Endurance sports<br>Candidate-gene association study	VEGFR2 rs2305948	Athletic performance or performance-related phenotypes	VEGFR2 rs2305948 genotype distribution did not differ significantly between athletes and controls; no effect on aerobic performance was demonstrated.
Y0016 (2025)<br>Effects of Exercise Addiction and the COL1A1 Gene rs1800012 Polymorphism on Injury Susceptibility in Elite Female Volleyball Players	POP-16<br>Confirmed shared cohort<br>Athletes 50; controls 557; total 607	Women's volleyball<br>Candidate-gene association study	COL1A1 rs1800012	Injury susceptibility	In Turkish female volleyball players, the COL1A1 T allele and exercise dependence were associated with injury; the GT genotype was associated with a longer recovery time.
Y0028 (2024)<br>Investigation of the putative functional relevance of the IL-6 3'UTR genetic variants with athletic phenotype in Turkish triathletes	POP-19<br>Single/independent<br>Athletes 47; controls 46; total 93	Triatlon<br>Candidate-gene association study	IL-6 3′UTR	Athletic performance or performance-related phenotypes	Four variants were identified in the IL6 region; rs13306436 was found only in triathletes and showed a potentially functional effect related to miRNA binding in silico.
Y0041 (2023)<br>The Relationship among the Performance and the ACE I/D and ACTN3 R577X Polymorphisms and some Anthropometric Factors in Arm Wrestlers.	POP-20<br>Single/independent<br>Athletes 47; controls 34; total 81	Arm wrestling<br>Candidate-gene association study	ACE I/D ve ACTN3 R577X	Physiological, metabolic, or anthropometric traits	ACE and ACTN3 polymorphisms did not differ significantly across the three groups; national athletes had larger palm, wrist, and forearm circumferences than sedentary participants.
Y0063 (2024)<br>The ARK2N (C18ORF25) Genetic Variant Is Associated with Muscle Fiber Size and Strength Athlete Status.	POP-02<br>Confirmed shared cohort<br>Athletes 60; controls 20; total 80	Track and field<br>Candidate-gene association study	ARK2N rs6507691	Athletic performance or performance-related phenotypes	No significant association of ARK2N rs6507691 with control status or competition performance was found in Turkish power and endurance athletes.
Y0089 (2023)<br>PPARA and IL6: exploring associations with athletic performance and genotype polymorphism.	POP-21<br>Single/independent<br>Athletes 30; controls 0; total 30	Cross-country skiing<br>Candidate-gene association study	IL6 ve PPARA	Athletic performance or performance-related phenotypes	The IL6 GC + PPARA GG combination was more prominent in the faster 1-km performance group; among men, the IL6 GC genotype and C allele were more frequent in the faster subgroup.
Y0100 (2024; 2024/08/01/Aug2024)<br>Association between uncoupling protein 1-3826 A/G polymorphism and the effects of anaerobic exercises on serum oxidative stress; [Relation entre le polymorphisme de la protéine découplante 1-3826 A/G et les effets des exercices anaérobies sur le stress oxydatif sérique]	POP-22<br>Single/independent<br>Athletes 43; controls 43; total 86	Anaerobic sports<br>Candidate-gene association study	UCP1 -3826 A/G	Physiological, metabolic, or anthropometric traits	Chronic anaerobic training reduced UCP-1 and was associated with higher oxidative stress in GG homozygous athletes.

Report	Population and sample	Sport and approach	Genetic marker(s)	Primary phenotypic theme	Main finding
Y0120 (2023)<br>The evaluation of superoxide dismutase 1 gene insertion/deletion variant in athletes	POP-23<br>Single/independent<br>Athletes 165; controls 140; total 305	Çoklu elit spor<br>Candidate-gene association study	SOD1 insersiyon/delesyon	Athlete status/participation or genotype distribution	The SOD1 I allele and I/I genotype were more frequent in female athletes than controls; no athlete-control difference was detected among men.
Y0127 (2025)<br>Genome-Wide Association Study of Exercise Addiction Among Elite Wrestlers	POP-01<br>Confirmed shared cohort<br>Athletes 67; controls 0; total 67	Wrestling<br>Genome-wide association study (GWAS)	Genome-wide variants	Sport-related psychological traits	Suggestive associations of six SNPs with exercise dependence were detected; the findings were hypothesis-generating because of the small sample.
Y0131 (2024)<br>Exploring the relationship between caffeine metabolism-related CYP1A2 rs762551 polymorphism and team sport athlete status and training adaptations	POP-24<br>Single/independent<br>Athletes 25; controls 25; total 50	Basketbol<br>Candidate-gene association study	CYP1A2 rs762551	Athletic performance or performance-related phenotypes	The CYP1A2 rs762551 CC genotype was reported less frequently among elite team-sport athletes; training response was also assessed in a Turkish basketball subgroup.
Y0203 (2024)<br>Evaluation of a circadian rhythm gene (PER3) VNTR variant in Turkish athletes	POP-25<br>Single/independent<br>Athletes 123; controls 100; total 223	Multiple sports<br>Candidate-gene association study	PER3 VNTR	Athlete status/participation or genotype distribution	Overall genotype and allele distributions for the PER3 VNTR were not significant; an athlete-control association was reported under a recessive model.
Y0209 (2025)<br>Genetic biomarkers of skiers from the Oğlağo Tribe in Muş Province, Turkey: An analysis of ACTN3, VEGF-A, and GABPB1 polymorphisms.	POP-26<br>Single/independent<br>Athletes 19; controls 130; total 149	Kayak<br>Candidate-gene association study	ACTN3, VEGF-A, GABPB1	Athlete status/participation or genotype distribution	There was no significant group difference for ACTN3 or VEGF-A; GABPB1 genotype and allele distributions differed significantly between skier and control groups.
Y0213 (2024)<br>Evaluation of the 18-bp deletion/insertion variant of the VEGF gene in athletes.	POP-27<br>Single/independent<br>Athletes 146; controls 170; total 316	Multiple sports<br>Candidate-gene association study	VEGF insersiyon/delesyon	Athlete status/participation or genotype distribution	The VEGF D allele and D/D genotype were more frequent in athletes than controls; no significant difference was detected across sports.
Y0221 (2022)<br>ACTN3 R577X variant: could it be a determinant of sports performance in elite athletes in a Turkish population? A. Canikli et al.	POP-28<br>Single/independent<br>Athletes 168; controls 148; total 316	Çoklu elit spor<br>Candidate-gene association study	ACTN3 R577X	Athletic performance or performance-related phenotypes	ACTN3 genotype distribution differed between elite athletes and controls; no significant difference in allele frequencies was reported.
Y0264 (2022)<br>Comparison of potential biomarker, ACTN3 rs1815739 polymorphism, for athletic performance of Turkish athletes.	POP-29<br>Single/independent<br>Athletes 131; controls 89; total 220	Multiple sports<br>Candidate-gene association study	ACTN3 rs1815739	Athlete status/participation or genotype distribution	The ACTN3 RR genotype was more frequent in sprint/distance runners; RR/RX distributions were similar in cyclists, bodybuilders, and Ironman groups.
Y0284 (2023)<br>Evaluation of the Association of VDR rs2228570 Polymorphism with Elite Track and Field Athletes' Competitive Performance.	POP-02<br>Confirmed shared cohort<br>Athletes 60; controls 20; total 80	Track and field<br>Candidate-gene association study	VDR rs2228570	Athletic performance or performance-related phenotypes	No significant association was detected between VDR rs2228570 and elite athlete status or personal-best competition performance.

Report	Population and sample	Sport and approach	Genetic marker(s)	Primary phenotypic theme	Main finding
Y0310 (2025)<br>Genetic Factors of Elite Wrestling Status: A Multi-Ethnic Comparative Study.	POP-01<br>Confirmed shared cohort<br>Athletes 67; controls 137; total 204	Wrestling<br>Genome-wide association study (GWAS)	Genome-wide variants	Athlete status/participation or genotype distribution	Across Turkish, Japanese, and Russian cohorts, ATP2A3 and UNC5C variants showed consistent associations with elite wrestler status; ACE rs4341 was also associated in the Turkish cohort.
Y0320 (2022)<br>EFFECTS OF GENETIC VARIATIONS OF MLCK2, AMPD1, AND COL5A1 ON MUSCLE ENDURANCE; [EFEITOS DAS VARIAÇÕES GENÉTICAS DE MLCK2, AMPD1 E COL5A1 SOBRE A RESISTÊNCIA MUSCULAR]; [EFECTOS DE LAS VARIACIONES GENÉTICAS DE MLCK2, AMPD1 Y COL5A1 SOBRE LA RESISTENCIA MUSCULAR]	POP-30<br>Single/independent<br>Athletes 33; controls 0; total 33	Soccer<br>Candidate-gene association study	MLCK2, AMPD1, COL5A1	Athletic performance or performance-related phenotypes	MLCK2, AMPD1, and COL5A1 variants were associated with some anthropometric and lower-extremity endurance measures.
Y0359 (2025)<br>Association analysis of indel variants and gene expression identifies <i>MDM4</i> as a novel locus for skeletal muscle hypertrophy and power athlete status	POP-02<br>Confirmed shared cohort<br>Athletes 60; controls 20; total 80	Track and field<br>Targeted inherited indel-variant study	MDM4/indel variants	Athlete status/participation or genotype distribution	The MDM4 rs35493922 D allele was overrepresented in Turkish power athletes and was associated with a muscle-hypertrophy/fast-twitch-fibre phenotype.
Y0364 (2025)<br>Association of ANKK1 and DRD2 gene polymorphisms with exercise addiction among elite athletes	POP-31<br>Single/independent<br>Athletes 107; controls 0; total 107	Badminton ve güreş<br>Candidate-gene association study	ANKK1/DRD2	Sport-related psychological traits	Four SNPs in the dopaminergic ANKK1/DRD2 region were associated with exercise dependence; replication is required.
Y0402 (2023)<br>Association of ACTN3 R577X Polymorphism with Elite Basketball Player Status and Training Responses	POP-32<br>Single/independent<br>Athletes 20; controls 47; total 67	Basketbol<br>Candidate-gene association study	ACTN3 R577X	Athletic performance or performance-related phenotypes	The ACTN3 XX genotype was less frequent in elite basketball players; the RR genotype was associated with the greatest physical-performance response to 6 weeks of training.
Y0414 (2023)<br>The Distinguishing Factor in Soccer Players is Aerobic Performance, not the ACTN3 Gene; [El Factor Distintivo en los Jugadores de Fútbol es el Rendimiento Aeróbico y no el gen ACTN3]	POP-33<br>Single/independent<br>Athletes 133; controls 0; total 133	Soccer<br>Candidate-gene association study	ACTN3 R577X	Athletic performance or performance-related phenotypes	Aerobic performance was higher in professional football players; ACTN3 rs1815739 distribution did not differ significantly between groups.
Y0454 (2024)<br>Comparison of MCM6 rs4988235 Polymorphism Allele and Genotype Frequencies in Professional Football Players and a Sedentary Control Group	POP-34<br>Single/independent<br>Athletes 20; controls 64; total 84	Soccer<br>Candidate-gene association study	MCM6 rs4988235	Athlete status/participation or genotype distribution	MCM6 rs4988235 genotype and allele frequencies did not differ significantly between football players and sedentary participants.
Y0464 (2023)<br>Exome-Wide Association Study of Competitive Performance in Elite Athletes.	POP-02<br>Confirmed shared cohort<br>Athletes 60; controls 20; total 80	Track and field<br>Exome-wide approach	Exome-wide variants	Athletic performance or performance-related phenotypes	No exome-wide significant SNP was detected in Turkish athletes; four functional variants were replicated in independent cohorts.

Report	Population and sample	Sport and approach	Genetic marker(s)	Primary phenotypic theme	Main finding
Y0468 (2025)<br>The ACTN3 R577X Nonsense Allele Is Underrepresented in Professional Volleyball Players and Associated with an Increased Risk of Muscle Injury in Female Players	POP-16<br>Confirmed shared cohort<br>Athletes 50; controls 557; total 607	Women's volleyball<br>Candidate-gene association study	ACTN3 R577X	Injury susceptibility	The ACTN3 X allele was less frequent in professional volleyball players; the XX genotype was associated with non-contact muscle injury in Turkish female volleyball players.
Y0482 (2024)<br>Sprint and Anaerobic Power with the Soccer-Specific ACTN3 Gene: A Distintive Example; [Sprint y Potencia Anaeróbica con el Gen ACTN3 Específico del Fútbol: Un Ejemplo Distintivo]	POP-35<br>Single/independent<br>Athletes 133; controls 0; total 133	Soccer<br>Candidate-gene association study	ACTN3 R577X	Athletic performance or performance-related phenotypes	ACTN3 polymorphism did not distinguish professional from amateur football players; sprint and anaerobic-power parameters were discriminative.
Y0486 (2024)<br>The AMPD1 Gene's rs17602729 Polymorphism and Athletic Performance in Track and Field Athletes	POP-02<br>Confirmed shared cohort<br>Athletes 60; controls 20; total 80	Track and field<br>Candidate-gene analysis using exome data	AMPD1 rs17602729	Athletic performance or performance-related phenotypes	The AMPD1 rs17602729 variant was examined in relation to athlete status and competition performance in elite Turkish athletes; exact effect estimates required confirmation during second extraction.
Y0511 (2019; 2019/01/01/2019)<br>The relationship between ACTN3 R577X gene polymorphism and physical performance in amateur soccer players and sedentary individuals.	POP-36<br>Single/independent<br>Athletes 100; controls 101; total 201	Soccer<br>Candidate-gene association study	ACTN3 R577X	Athletic performance or performance-related phenotypes	ACTN3 R577X distribution was similar between football players and controls; the variant was not a sufficiently specific marker of sprint/endurance in amateur football players.
Y0512 (2020)<br>Distribution of α-actinin-3 rs1815739 and angiotensin-1 converting enzyme InDel polymorphisms in Turkish bodybuilders	POP-37<br>Single/independent<br>Athletes 11; controls 0; total 11	Bodybuilding<br>Candidate-gene association study	ACTN3 ve ACE	Athletic performance or performance-related phenotypes	ACE ID and ACTN3 RX were the most frequent genotypes in Turkish bodybuilders; the finding was descriptive because no control group was included.
Y0612 (2021)<br>A Case-Control Study Investigating the Effect of MTHFR C677T Variant on Performance of Elite Athletes.	POP-38<br>Single/independent<br>Athletes 114; controls 100; total 214	Çoklu elit spor<br>Candidate-gene association study	MTHFR C677T	Athletic performance or performance-related phenotypes	The MTHFR C677T CC genotype and C allele were more frequent in elite Turkish athletes, whereas the CT genotype was more frequent in controls.
Y0649 (2020)<br>Peroxisome Proliferator-Activated Receptor Alpha (PPARα) rs4253778 Polymorphism in a Turkish Soccer Player Cohort	POP-39<br>Single/independent<br>Athletes 22; controls 68; total 90	Soccer<br>Candidate-gene association study	PPARA rs4253778	Athlete status/participation or genotype distribution	The PPARA GG genotype (63.6%) and G allele (81.8%) were more frequent in professional football players than controls.
Y1006 (2023)<br>Futbolcularda ACTN3 rs1815739 Polimorfizmi, Fiziksel Özellikler ve Mevki İlişkisinin Araştırılması: Bir Takım Örneklemi	POP-40<br>Single/independent<br>Athletes 20; controls 76; total 96	Soccer<br>Candidate-gene association study	ACTN3 rs1815739	Athletic performance or performance-related phenotypes	CC/CT/TT distributions in football players were reported; the source table required confirmation by a second extractor for positional associations.

Report	Population and sample	Sport and approach	Genetic marker(s)	Primary phenotypic theme	Main finding
Y1007 (2022)<br>Evaluation of association between PPARGC1A gene polymorphism and competitive performance of elite athletes	POP-02<br>Confirmed shared cohort<br>Athletes 60; controls 20; total 80	Track and field<br>Candidate-gene association study	PPARGC1A	Athletic performance or performance-related phenotypes	No significant association with personal-best score was found across genotype groups or within sprint/power-endurance subgroups (p>0.05).
Y0975 (2023; 2023/03/01/Mar2023)<br>ASSOCIATION BETWEEN MCT1 GENE POLYMORPHISM (rs1049434) WITH THE ATHLETIC PERFORMANCE OF ELITE TRACK AND FIELD ATHLETES.	POP-02<br>Confirmed shared cohort<br>Athletes 60; controls 20; total 80	Track and field<br>Candidate-gene association study	MCT1	Athletic performance or performance-related phenotypes	In the endurance group, personal-best score was associated with rs1049434 under codominant (p=0.044), dominant (p=0.016), and over-dominant (p=0.048) models; no association was found in the sprint/power group.
Y1009 (2021)<br>FUTBOLCULARDA SLC6A4 PROMOTÖR POLİMORFİZMİNİN DAĞILIMI	POP-41<br>Single/independent<br>Athletes 19; controls 45; total 64	Soccer<br>Candidate-gene association study	SLC6A4	Athlete status/participation or genotype distribution	Athlete-control genotype distribution was significant (p=0.0001), as was allele distribution (p=0.0425). In football players, LL was 57.8% and the L allele was 68.4%.
Y1028 (2021; 2023)<br>Distribution of dopamine receptor 2 DRD2 rs1800497 polymorphisms in professional football players	POP-42<br>Single/independent<br>Athletes 21; controls 52; total 73	Soccer<br>Candidate-gene association study	DRD2	Athlete status/participation or genotype distribution	The DRD2 variant was assessed in football players and controls.
Y1001 (2023)<br>Athletic performance, sports experience, and exercise addiction: An association study on ANKK1 gene polymorphism rs1800497	POP-02<br>Confirmed shared cohort<br>Athletes 60; controls 20; total 80	Track and field<br>Candidate-gene association study	ANKK1	Sport-related psychological traits	ANKK1 was examined in a Turkish Athletics Federation sample.
Y1017 (2019)<br>Profesyonel Voleybolcularda Katekol-O-Metiltransferaz (COMT) rs4680 Polimorfizminin Belirlenmesi	POP-43<br>Single/independent<br>Athletes 21; controls 0; total 21	Voleybol<br>Candidate-gene association study	COMT	Athlete status/participation or genotype distribution	COMT rs4680 genotype and allele distributions were described in professional volleyball players.
Y0895 (2026)<br>Genome-Wide Association Study of Vertical Jump Performance Among Elite Badminton Players	POP-44<br>Single/independent<br>Athletes 90; controls 557; total 647	Badminton<br>Genome-wide association study (GWAS)	Genome-wide variants	Athletic performance or performance-related phenotypes	Genomic signals associated with the vertical-jump phenotype were assessed.
Y1043 (2021)<br>Collagen type 1 gene (COL1A1) rs1800012 polymorphism in long and short distance runners.	POP-45<br>Single/independent<br>Athletes 44; controls 50; total 94	Running<br>Candidate-gene association study	COL1A1	Injury susceptibility	Genotype comparisons were non-significant for long- versus short-distance athletes (p=0.870), short-distance athletes versus controls (p=0.510), and long-distance athletes versus controls (p=0.201).
Y1013 (2021)<br>Analysis of HIF1A (rs11549465) Polymorphism Distributions in Female Volleyball Players	POP-46<br>Single/independent<br>Athletes 21; controls 0; total 21	Women's volleyball<br>Candidate-gene association study	HIF1A	Athlete status/participation or genotype distribution	CC 16 (%76.2), CT 5 (%23.8), TT 0; C alleli %88.1 ve T alleli %11.9.

Report	Population and sample	Sport and approach	Genetic marker(s)	Primary phenotypic theme	Main finding
Y1033 (2021)<br>Investigation of Alpha-Actinin-3 (ACTN3) Gene Polymorphism in Bodybuilding Athletes: The Case of Samsun Province in Turkey	POP-47<br>Single/independent<br>Athletes 35; controls 0; total 35	Bodybuilding<br>Candidate-gene association study	ACTN3	Athlete status/participation or genotype distribution	RR/RX/XX distribution did not differ significantly between national and non-national bodybuilders ($p>0.05$).
Y1020 (2022)<br>The Determination Of The Relationship Between Vo2max And The Angiotensin-Converting Enzyme Gene (ACE) rs1799752 Polymorphisms In The Turkish National Ice Hockey Sports Team	POP-48<br>Single/independent<br>Athletes 21; controls 0; total 21	Ice hockey<br>Candidate-gene association study	ACE I/D	Athletic performance or performance-related phenotypes	II/ID/DD counts were 9/7/5, respectively; mean VO_2max values were 49.74/47.34/46.43 $mL\cdot kg^{-1}\cdot min^{-1}$. The difference was not significant ($p>0.05$).
Y0983 (2021)<br>Futbolcularda Anjiyotensinojen (AGT) rs699 Polimorfizm Dağılımının Belirlenmesi	POP-49<br>Single/independent<br>Athletes 52; controls 106; total 158	Soccer<br>Candidate-gene association study	AGT	Athlete status/participation or genotype distribution	Genotype $\chi^2=1.774$, $p=0.438$; allele $\chi^2=0.993$, $p=0.465$. There was no significant difference between groups.
Y1012 (2020)<br>Türk ironman triatlon sporcularında IL-6, HIF1A, MCT1, PPAR-a Polimorfizm dağılımının belirlenmesi	POP-50<br>Single/independent<br>Athletes 10; controls 0; total 10	Ironman<br>Candidate-gene association study	IL6/HIF1A/MCT1/PPARA	Athlete status/participation or genotype distribution	PPARA GG=9/10; MCT1 AA=9/10; HIF1A CC=7/10; IL6 GG=6/10. Kontrol grubu yoktur.
MAN-01 (2024)<br>ACTN3 R577X Polymorphism and Anaerobic Performance in Ultimate Frisbee Players: A Preliminary Study	POP-51<br>Single/independent<br>Athletes 30; controls 30; total 60	Ultimate Frisbee<br>Candidate-gene association study	ACTN3 R577X	Athletic performance or performance-related phenotypes	The association between ACTN3 R577X and anaerobic performance was examined.
MAN-02 (2025)<br>Investigation of the Effects of ACTN3 rs1815739, PPARA rs4253778 and IL6 rs1800795 Gene Polymorphism on Aerobic Endurance in Volleyball Players	POP-52<br>Single/independent<br>Athletes 22; controls 0; total 22	Voleybol<br>Candidate-gene association study	ACTN3/PPARA/IL6	Athletic performance or performance-related phenotypes	Associations of three candidate genes with aerobic endurance were examined.
MAN-03 (2023)<br>Investigation of the ACTN3 Gene Polymorphism (rs1815739) and FMS Values in Young Runners: Cross-Sectional Research	POP-53<br>Single/independent<br>Athletes 45; controls 0; total 45	Running<br>Candidate-gene association study	ACTN3 rs1815739	Injury susceptibility	The association between ACTN3 rs1815739 and FMS scores was examined.
MAN-04 (2023)<br>The relationship between the SLC6A4 gene polymorphism (rs 5-HTTLPR) and aggression in combat athletes	POP-10<br>Confirmed shared cohort<br>Athletes 40; controls 0; total 40	Combat sports<br>Candidate-gene association study	SLC6A4 5-HTTLPR	Sport-related psychological traits	The association between 5-HTTLPR and aggression was examined.
MAN-05 (2024)<br>Determination of COL1A1 rs1800012 Polymorphism Related to Ligament and Tendon Injury in Turkish Professional Bodybuilders: Experimental Research	POP-54<br>Single/independent<br>Athletes 15; controls 50; total 65	Bodybuilding<br>Candidate-gene association study	COL1A1 rs1800012	Injury susceptibility	The association between COL1A1 rs1800012 and injury was examined.
MAN-06 (2022)<br>Türk Uzun ve Kısa Mesafe Koşucularında Anjiotensinojen Geni (rs699) Polimorfizminin Atletik Performans Üzerine Etkisi	POP-55<br>Single/independent<br>Athletes 24; controls 0; total 24	Running<br>Candidate-gene association study	AGT rs699	Athletic performance or performance-related phenotypes	The association between AGT rs699 and athletic performance was examined.

Report	Population and sample	Sport and approach	Genetic marker(s)	Primary phenotypic theme	Main finding
MAN-07 (2023)<br>Investigation of Vitamin D Receptor Gene rs731236 Polymorphism in Turkish Rugby Players	POP-56<br>Single/independent<br>Athletes 22; controls 30; total 52	Rugby<br>Candidate-gene association study	VDR rs731236	Athlete status/participation or genotype distribution	VDR rs731236 distribution was examined in rugby players and controls.
MAN-08 (2024)<br>Futsal ve Futbolcuların SLC6A4 Geni 5-HTTLPR Dağılımı	POP-57<br>Single/independent<br>Athletes 48; controls 0; total 48	Futsal/soccer<br>Candidate-gene association study	SLC6A4 5-HTTLPR/rs25531	Athlete status/participation or genotype distribution	SLC6A4 5-HTTLPR distribution was assessed in futsal and football players.
MAN-09 (2025)<br>Examination of the Relationship Between ACE rs4343 Gene Polymorphism and Yo-Yo Intermittent Recovery Test (Level 2) Performance in Non-Elite Athletes	POP-58<br>Single/independent<br>Athletes 53; controls 0; total 53	Basketbol/voleybol<br>Candidate-gene association study	ACE rs4343	Athletic performance or performance-related phenotypes	The association between ACE rs4343 and Yo-Yo IR2 performance was examined.

Note. Sample sizes are reported at report level. Unique participant totals were calculated separately across 58 operationally unique study populations after confirmed cohort merging.

References for included reports

Each reference is linked to the Report ID used in Supplementary Table S1 and in the screening and extraction dataset.

Y0956. Kurtuluş, M., Günay, M., Çelenk, Ç., Cicioğlu, İ., & Kesici, T. (2019). Elit Türk güreşçilerinin anjiotensin i-dönüştürücü enzim gen polimorfizmi ve vo2max düzeyleri ile atletik performans arasindaki ilişkinin belirlenmesi. Spormetre Beden Eğitimi ve Spor Bilimleri Dergisi, 17(4), 275-284. doi:10.33689/spormetre.641112.

Y0965. Kurtuluş, M., Keskin, K., & Gunay, M. (2025). THE EVALUATION OF ACTN3 R577X POLYMORPHISM IN ELITE MALE TURKISH ATHLETES. Beden Eğitimi ve Spor Bilimleri Dergisi, 19(3), 330-340. doi:10.61962/bsd.1799101.

Y0970. BOZYURT, F., & YAMAK, B. (2024). Investigation of PPAR-Alpha Gene Polymorphism in Female's Soccer Players. Journal of ROL Sports Sciences, 5(4). 10.70736/jrolss.491.

Y0981. Kurtuluş, M., Keskin, K., Gunay, M., Kesici, T., & Gökdemir, K. (2023). Genetic differences in peroxisome proliferator-activated receptor alpha gene in endurance athletes (long distance runners) and power/endurance athletes (wrestlers, football players). Journal of Basic and Clinical Health Sciences, 7(2), 723-730. doi:10.30621/jbachs.1191220.

Y0984. Aslan, B. T., Yozgatlı, D. B., Yılmaz, Ö. Ö., Polat, T., Ekmekçi, R., & Ulucan, K. (2024). Distribution of Alpha-Actinin-3 (ACTN3) rs1815739 Polymorphism in HADO Athletes. Eurasian Research in Sport Science, 9(2), 64-71. https://izlik.org/JA85ZS35SR.

Y0992. Bulgay, C., Doğan, C. S., Karapınar, G., Polat, T., Cetın, E., & Ulucan, K. (2021). Farklı Branşlardaki Sporcuların Kollajen Tip V gen (COL5A1) rs12722 Polimorfizmlerinin Dağılımı. Gaziantep Üniversitesi Spor Bilimleri Dergisi, 6(1), 63-74. doi:10.31680/gaunjss.793223.

Y1003. Eken, B. F., Yılmaz, Ö. Ö., Polat, T., Tacal Aslan, B., & Ulucan, K. (2021). Türk Futbolcularda Alfa- Aktinin-3 (ACTN3) Ve Anjiyotensin Dönüştürücü Enzim (ACE) Polimorfizmleri Atletik Performans için Bir Biyobelirteç Olabilir mi? Eurasian Research in Sport Science, 6(2), 147-159. https://izlik.org/JA33KA78YU.

Y1021. Yiğit, S., Polat, T., Doğan, C. S., Tunalı, S., Ekmekçi, R., & Ulucan, K. (2020). PROFESYONEL VOLEYBOLCULARDA SLC6A4 PROMOTÖR L/S POLİMORFİZMİNİN BELİRLENMESİ VE LİTERATÜR KARŞILAŞTIRILMASI. The Journal of Neurobehavioral Sciences, 7(2), 65–69. doi:10.4103/JNBS.JNBS_8_20.

Y1026. Bilen, E. (2021). Mücadele sporcularında SLC6A4 geni 5-HTTLPR polimorfizmi ile saldırganlık düzeyleri arasındaki ilişkinin incelenmesi. Yüksek Lisans Tezi. Ondokuz Mayıs Üniversitesi, Lisansüstü Eğitim Enstitüsü, Samsun.

Y1029. Usaç, G., EROĞLU, O., & Zileli, R. (2020). The evaluation of rs1805086 and rs1805065 polymorphisms in mstn gene and anthropometric properties of national and amateur arm wrestlers Evaluación de los polimorfismos rs1805086 y rs1805065 en el gen mstn y las propiedades antropométricas de los luchadores de brazo nacionales y amateurs. International Journal of Morphology, 38(4). 10.4067/s0717-95022020000401148.

Y1032. Kaynar, Ö., Bilici, M. F., Sercan, C., & Ulucan, K. (2021). THE EXAMINATION OF THE EFFECTS OF PPAR-A (RS4253778) ON SERUM LIPIDS IN ELITE CROSS-COUNTRY SKIERS. Spor Ve Performans Araştırmaları Dergisi, 12(1), 72-79. doi:10.17155/omuspd.684940.

Y1037. Aslan, H. E. (2019). Kas dokusu fizyolojisinde rol oynayan iki aday gen varyantının alt ekstremite kas enduransı yönünden farklı mevkilerde oynayan futbolcularda incelenmesi. Yüksek Lisans Tezi, İstanbul Gelişim Üniversitesi Lisansüstü Eğitim Enstitüsü.

Y1041. Eken, B. F., Gezmış, H., Ük, Y., Erdoğdu, A., Doğan, C. S., Kıraç, D., & Ulucan, K. (2019). Türk Kökenli Futbolcularda Apolipoprotein-E Genotiplerinin Belirlenmesi. Eurasian Research in Sport Science, 4(2), 53-58. doi:10.35333/ERISS.2019.89.

Y1064. Kaynar, Ö., Bilici, F., Ulucan, K., & Sercan, C. (2019). Determination of HIF1Ars11549465 Polymorphism in Elite Skiers. Turkish Journal of Sport and Exercise, 21(2), 328-331. doi:10.15314/tsed.544566.

Y1092. Ercan, E., & Zulfinaz, C. (2020). VEGFR2 gene polymorphism and aerobic performance in Turkish Elite endurance athletes. INTERNATIONAL JOURNAL OF HUMAN GENETICS, (3). doi:10.31901/24566330.2020/20.03.759.

Y0016. Piri, M. M., Cerit, M., Anılır, M., Polat, T., Karaduman, A. A., Szabo, A., Antoun, T. G. A., John, G., Semenova, E. A., Larin, A. K., Kulemin, N. A., Generozov, E. V., & Ahmetov, I. I. (2025). Effects of Exercise Addiction and the COL1A1 Gene rs1800012 Polymorphism on Injury Susceptibility in Elite Female Volleyball Players. Genes, 16(11), 1300. doi:10.3390/genes16111300.

Y0028. Pirim, D., Niş, H. F., & Bağcı, F. A. (2024). Investigation of the putative functional relevance of the IL-6 3’UTR genetic variants with athletic phenotype in Turkish triathletes. Trakya University Journal of Natural Sciences, 25(2), 151-160. doi:10.23902/trkjnat.1493225.

Y0041. Zileli, R., Eroğlu, O., Nalbant, M. A., & Ulucan, K. (2023). The Relationship among the Performance and the ACE I/D and ACTN3 R577X Polymorphisms and some Anthropometric Factors in Arm Wrestlers. Cellular and molecular biology (Noisy-le-Grand, France), 69(5), 1–5. doi:10.14715/cmb/2023.69.5.1.

Y0063. Çığırtaş, R., Bulgay, C., Kazan, H. H., Akman, O., Sporiš, G., John, G., Yusupov, R. A., Sultanov, R. I., Zhelankin, A. V., Semenova, E. A., Larin, A. K., Kulemin, N. A., Generozov, E. V., Jurko, D., & Ahmetov, I. I. (2024). The ARK2N (C18ORF25) Genetic Variant Is Associated with Muscle Fiber Size and Strength Athlete Status. Metabolites, 14(12), 684. doi:10.3390/metabo14120684.

Y0089. Kazancı, D., Polat, T., Kaynar, Ö., Bilici, M. F., Tacal Aslan, B., & Ulucan, K. (2023). PPARA and IL6: exploring associations with athletic performance and genotype polymorphism. Cellular and molecular biology (Noisy-le-Grand, France), 69(11), 69–75. doi:10.14715/cmb/2023.69.11.12.

Y0100. Chauchat, J. R., Turgay, F., Kırmızıgil, B., Yigittürk, O., Durmaz, M. B., & Aşıkovalı, S. (2024). Association between uncoupling protein 1-3826 A/G polymorphism and the effects of anaerobic exercises on serum oxidative stress. Science & Sports, 39(5-6), 472-482. doi:10.1016/j.scispo.2023.04.009.

Y0120. Nursal, A. F., Ünver, Ş., Yiğit, S., Orhan, Ö. M., & Atan, T. (2023). The evaluation of superoxide dismutase 1 gene insertion/deletion variant in athletes. Revista da Associação Médica Brasileira, 69(12), e20230575. doi:10.1590/1806-9282.20230575.

Y0127. Bulgay, C., Kasakolu, A., Bıyıklı, T., Koncagul, S., Kazan, H. H., Ahmetov, I. I., Ergun, M. A., Griffiths, M. D., & Szabo, A. (2025). Genome-Wide Association Study of Exercise Addiction Among Elite Wrestlers. Brain Sciences, 15(2), 102. doi:10.3390/brainsci15020102.

Y0131. Kazan, H. H., Bulgay, C., Zorba, E., Dalip, M., Ceylan, H. İ., Semenova, E. A., ... & Cerit, M. (2024). Exploring the relationship between caffeine metabolism-related CYP1A2 rs762551 polymorphism and team sport athlete status and training adaptations. Molecular Biology Reports, 51(1), 841. doi:10.1007/s11033-024-09800-2.

Y0203. Unver, S., Yigit, S., Tural, E., Yigit, E., & Atan, T. (2024). Evaluation of a circadian rhythm gene (PER3) VNTR variant in Turkish athletes. Nucleosides, Nucleotides & Nucleic Acids, 43(2), 173-183. doi:10.1080/15257770.2023.2248198.

Y0209. Kaynar, Ö., Polat, T., Koç, H., Ferhatoğlu, Y., Yilmaz, Ö. Ö., Tacal Aslan, B., Ulucan, K., & Gómez-Salgado, J. (2025). Genetic biomarkers of skiers from the Oğlağo Tribe in Muş Province, Turkey: An analysis of ACTN3, VEGF-A, and GABPB1 polymorphisms. Medicine, 104(20), e42429. doi:10.1097/MD.0000000000042429.

Y0213. Unver, S., Nursal, A. F., Yigit, S., & Atan, T. (2024). Evaluation of the 18-bp deletion/insertion variant of the VEGF gene in athletes. Nucleosides, Nucleotides & Nucleic Acids, 43(11), 1252–1261. doi:10.1080/15257770.2024.2304145.

Y0221. Canikli, A., et al. (2022). ACTN3 R577X variant: could it be a determinant of sports performance in elite athletes in a Turkish population? Journal of Genetics, 101(1), Article 30. doi:10.1007/s12041-022-01362-1.

Y0264. Dogan, M., Tacal Aslan, B., & Ulucan, K. (2022). Comparison of potential biomarker, ACTN3 rs1815739 polymorphism, for athletic performance of Turkish athletes. Cellular and Molecular Biology, 68(5), 54–59. doi:10.14715/cmb/2022.68.5.7.

Y0284. Bulgay, C., Bayraktar, I., Kazan, H. H., Yıldırım, D. S., Zorba, E., Akman, O., Ergun, M. A., Cerit, M., Ulucan, K., Eken, Ö., Ceylan, H. İ., Badicu, G., Grosz, W. R., & Mijaică, R. (2023). Evaluation of the association of VDR rs2228570 polymorphism with elite track and field athletes' competitive performance. Healthcare, 11(5), Article 681. doi:10.3390/healthcare11050681.

Y0310. Kozuma, A., Bulgay, C., Zempo, H., Saito, M., Deguchi, M., Homma, H., Matsumoto, S., Matsumoto, R., Kasakolu, A., Kazan, H. H., Bıyıklı, T., Koncagül, S., Baydaş, G., Ergun, M. A., Szabo, A., Semenova, E. A., Larin, A. K., Kulemin, N. A., Generozov, E. V., … Kikuchi, N. (2025). Genetic factors of elite wrestling status: A multi-ethnic comparative study. Genes, 16(8), Article 906. doi:10.3390/genes16080906.

Y0320. Horozoglu, C., Aslan, H. E., Karaagac, A., Kucukhuseyin, O., Bilgic, T., Himmetoglu, S., Gheybi, A., Yaylim, I., & Zeybek, U. (2022). Effects of genetic variations of MLCK2, AMPD1, and COL5A1 on muscle endurance. Revista Brasileira de Medicina do Esporte, 28(4), 261–266. doi:10.1590/1517-8692202228022021_0180.

Y0359. Kazan, H. H., Kasakolu, A., Koncagul, S., Ergun, M. A., John, G., Sultanov, R. I., Zhelankin, A. V., Semenova, E. A., Yusupov, R. A., Kulemin, N. A., Larin, A. K., Generozov, E. V., Bulgay, C., & Ahmetov, I. I. (2025). Association analysis of indel variants and gene expression identifies MDM4 as a novel locus for skeletal muscle hypertrophy and power athlete status. Experimental Physiology, 110(11), 1661–1671. doi:10.1113/EP091992.

Y0364. Yıldız, Y. A., Kasakolu, A., Bulgay, C., Bıyıklı, T., Kazan, H. H., Koncagul, S., Bayraktar, I., Ahmetov, I. I., Griffiths, M. D., Ergun, M. A., & Szabo, A. (2025). Association of ANKK1 and DRD2 gene polymorphisms with exercise addiction among elite athletes. International Journal of Sport and Exercise Psychology. Advance online publication. doi:10.1080/1612197X.2025.2584537.

Y0402. Demirci, B., Bulgay, C., Ceylan, H. İ., Öztürk, M. E., Öztürk, D., Kazan, H. H., Ergun, M. A., Cerit, M., Semenova, E. A., Larin, A. K., Generozov, E. V., Ahmetov, I. I., & Cepicka, L. (2023). Association of ACTN3 R577X polymorphism with elite basketball player status and training responses. Genes, 14(6), Article 1190. doi:10.3390/genes14061190.

Y0414. Zileli, R., Söyler, M., Diker, G., Özkamçı, H., Bayrakdaroglu, S., Sever, M. O., Can, I., Ön, S., Aslan, B. T., Ulucan, K., Polat, T., & Yılmaz, Ö. Ö. (2023). The distinguishing factor in soccer players is aerobic performance, not the ACTN3 gene. International Journal of Morphology, 41(5), 1564–1569. doi:10.4067/S0717-95022023000501564.

Y0454. Kanlı, S., & Çolak, R. (2024). Comparison of MCM6 rs4988235 polymorphism allele and genotype frequencies in professional football players and a sedentary control group. Sakarya University Journal of Science, 28(4), 815–823. doi:10.16984/saufenbilder.1500586.

Y0464. Bulgay, C., Kasakolu, A., Kazan, H. H., Mijaica, R., Zorba, E., Akman, O., Bayraktar, I., Ekmekci, R., Koncagul, S., Ulucan, K., Semenova, E. A., Larin, A. K., Kulemin, N. A., Generozov, E. V., Balint, L., Badicu, G., Ahmetov, I. I., & Ergun, M. A. (2023). Exome-wide association study of competitive performance in elite athletes. Genes, 14(3), Article 660. doi:10.3390/genes14030660.

Y0468. Cerit, M., Tuncer, S. Y., Piri, M. M., Anılır, M., John, G., Semenova, E. A., Larin, A. K., Generozov, E. V., Ahmetov, I. I., Ulucan, K., & Szabo, A. (2025). The ACTN3 R577X nonsense allele is underrepresented in professional volleyball players and associated with an increased risk of muscle injury in female players. Genes, 16(9), Article 1076. doi:10.3390/genes16091076.

Y0482. Söyler, M., Zileli, R., Özkamçı, H., Diker, G., Sever, M. O., Bayrakdaroğlu, S., Ön, S., Can, I., Ulucan, K., Aslan, B. T., Polat, T., & Yılmaz, Ö. Ö. (2024). Sprint and anaerobic power with the soccer-specific ACTN3 gene: A distinctive example. International Journal of Morphology, 42(2), 538–548. doi:10.4067/S0717-95022024000200538.

Y0486. Bulgay, C., Çakır, V. O., Kazan, H. H., Ergün, M. A., Badicu, G., & Ardigò, L. P. (2024). The AMPD1 gene's rs17602729 polymorphism and athletic performance in track and field athletes. Applied Sciences, 14(2), Article 891. doi:10.3390/app14020891.

Y0511. Koku, F. E., Karamızrak, S. O., Çiftçi, A. S., Taşlıdere, H., Durmaz, B., & Çoğulu, Ö. (2019). The relationship between ACTN3 R577X gene polymorphism and physical performance in amateur soccer players and sedentary individuals. Biology of Sport, 36(1), 9–16. doi:10.5114/biolsport.2018.78900.

Y0512. Polat, T., Dogan, C. S., Dogan, M., Akçay, T., & Ulucan, K. (2020). Distribution of α-actinin-3 rs1815739 and angiotensin-1 converting enzyme InDel polymorphisms in Turkish bodybuilders. Biomedical Reports, 13(6), Article 67. doi:10.3892/br.2020.1374.

Y0612. Nursal, A. F., Yigit, S., Rustemoglu, H., & Cenikli, A. (2021). A case-control study investigating the effect of MTHFR C677T variant on performance of elite athletes. Endocrine, Metabolic & Immune Disorders - Drug Targets, 21(9), 1685–1690. doi:10.2174/1568026620666201022144819.

Y0649. Ulucan, K., Ük, Y., Kapıcı, S., Yüksel, İ., Sercan, C., & Eken, B. F. (2020). Peroxisome Proliferator-Activated Receptor Alpha (PPARα) rs4253778 Polymorphism in a Turkish Soccer Player Cohort. Pamukkale Journal of Sport Sciences, 11(1), 1-6. https://izlik.org/JA78LZ79TG.

Y1006. Muhan, A., Polat, T., Yılmaz, Ö. Ö., Tacal Aslan, B., & Ulucan, K. (2023). Futbolcularda ACTN3 rs1815739 Polimorfizmi, Fiziksel Özellikler ve Mevki İlişkisinin Araştırılması: Bir Takım Örneklemi. Research in Sport Education and Sciences, 25(1), 14-18. doi:10.5152/JPESS.2023.221840.

Y1007. Bulğay, C., Zorba, E., Akman, O., Bayraktar, I., Kazan, H. H., Ergun, M. A., & Ulucan, K. (2022). Evaluation of Association between PPARGC1A Gene Polymorphism and Competitive Performance of Elite Athletes. Gazi Journal of Physical Education and Sport Sciences, 27(4), 323-332. doi:10.53434/gbesbd.1126033.

Y0975. Bulğay, C., Zorba, E., Bayraktar, I., Kazan, H. H., Ulucan, K., & Ergun, M. A. (2023). Association between MCT1 gene polymorphism (rs1049434) with the athletic performance of elite track and field athletes. SPORMETRE Beden Eğitimi ve Spor Bilimleri Dergisi, 21(1), 127–134. doi:10.33689/spormetre.1198404.

Y1009. Eken, B. F., Polat, T., Sercan Doğan, C., Oktay, Ş., Tacal Aslan, B., & Ulucan, K. (2021). FUTBOLCULARDA SLC6A4 PROMOTÖR POLİMORFİZMİNİN DAĞILIMI. Spor Ve Performans Araştırmaları Dergisi, 12(3), 212-221. doi:10.17155/omuspd.959572.

Y1028. Tacal Aslan, B., Yılmaz, Ö. Ö., Polat, T., Şılar, Ç., Başak, F., Doğan, C. S., & Ulucan, K. (2021). Distribution of dopamine receptor 2 DRD2 rs1800497 polymorphisms in professional football players: A pilot study. Turkish Journal of Sport and Exercise, 23(2), 185–189. doi:10.15314/tsed.905202.

Y1001. Bayraktar I, Cepicka L, Barasinska M, Kazan HH, Zorba E, Ergun MA, Eken Ö, Ceylan Hİ, Bulgay C and Gabrys T (2023) Athletic performance, sports experience, and exercise addiction: an association study on ANKK1 gene polymorphism rs1800497. Front. Psychol. 14:1182575. doi:10.3389/fpsyg.2023.1182575.

Y1017. Tunalı, S., Yiğit, S., Sercan, C., Polat, T., Ekmekçi, R., & Ulucan, K. (2019). Profesyonel Voleybolcularda Katekol-O-Metiltransferaz (COMT) rs4680 Polimorfizminin Belirlenmesi. Eurasian Research in Sport Science, 4(1), 1-6. doi:10.22396/ERISS.2019153188.

Y0895. Sökmen, F. C., Kasakolu, A., Bulgay, C., Kikuchi, N., Kazan, H. H., Koncagul, S., Yildiz, Y. A., Szabo, A., Bragazzi, N. L., & Ergün, M. A. (2026). Genome-wide association study of vertical jump performance among elite badminton players. International Journal of Molecular Sciences, 27(6), Article 2533. doi:10.3390/ijms27062533.

Y1043. Bulğay, C., Doğan, C. S., Çetin, E., Polat, T., Eken, B. F., Akkoç, O., ... & Ulucan, K. (2021). Collagen type 1 gene (COL1A1) rs1800012 polymorphism in long and short distance runners. Spor Hekimligi Dergisi/Turkish Journal of Sports Medicine, 56(1), 28. doi. 10.47447/tjsm.0469.

Y1013. Gözler, İ. Y., Kavas, N. C., Polat, T., Doğan, C. S., Gözler, T., Aslan, B. T., & Ulucan, K. (2021). Analysis of HIF1A (rs11549465) Polymorphism Distributions in Female Volleyball Players. Research in Sports Science, 11(1), 14-18. doi:10.5152/rss.2021.21002.

Y1033. Yamak, B., Eliöz, M., Çebi, M., Demirhan, B., & Akbuğa, E. (2021). Investigation of Alpha-Actinin-3 (ACTN3) Gene Polymorphism in Bodybuilding Athletes: The Case of Samsun Province in Turkey. International Journal of Applied Exercise Physiology, 10(2), 156-165.

Y1020. Doğan, C. S., Polat, T., Akkoc, O., Kurudirek, M. İ., & Ulucan, K. (2022). The Determination Of The Relationship Between Vo2max And The Angiotensin-Converting Enzyme Gene (ACE) rs1799752 Polymorphisms In The Turkish National Ice Hockey Sports Team. Cellular and Molecular Biology, 68(10), 90-93. doi:10.14715/cmb/2022.68.10.14.

Y0983. Bulgay, C., Polat, T., Yılmaz, Ö. Ö., Aslan, B. T., Ergun, M. A., & Ulucan, K. (2021). Futbolcularda Anjiyotensinojen (AGT) rs699 Polimorfizm Dağılımının Belirlenmesi. Kilis 7 Aralık Üniversitesi Beden Eğitimi ve Spor Bilimleri Dergisi, 5(2), 145-153. https://izlik.org/JA86JZ53AH.

Y1012. Akkoç, O., Birlik, A., Sercan Doğan, C., Ulucan, K., & Kırandı, Ö. (2020). Türk İronman Triatlon Sporcularında IL-6, HIF1A, MCT1, PPAR-a Polimorfizm Dağılımının Belirlenmesi. Spor Eğitim Dergisi, 4(1), 1-7. https://izlik.org/JA57LU23PW.

MAN-01. Pekdaş, M. A., Subaşı, F., Güleç, S., Kocadal, O., & İsbir, T. (2024). ACTN3 R577X polymorphism and anaerobic performance in Ultimate Frisbee players: A preliminary study. Türk Fizyoterapi ve Rehabilitasyon Dergisi, 35(3), 297–305. doi:10.21653/tjpr.1362944.

MAN-02. Sever, M. O., Can, İ., Bayrakdaroğlu, S., Ön, S., Özkamçı, H., Diker, G., Söyler, M., Zileli, R., Yılmaz, Ö. Ö., Polat, T., Tacal Aslan, B., & Ulucan, K. (2025). Investigation of the effects of ACTN3 rs1815739, PPARA rs4253778 and IL6 rs1800795 gene polymorphism on aerobic endurance in volleyball players. Beden Eğitimi ve Spor Bilimleri Dergisi, 19(1), 17–27. doi:10.61962/bsd.1540001.

MAN-03. Bulgay, C., Bayraktar, I., Ödemiş, M., Yıldırım, D. S., Ergün, M. A., Cerit, M., & Ulucan, K. (2023). Investigation of the ACTN3 gene polymorphism (rs1815739) and FMS values in young runners: Cross-sectional research. Türkiye Klinikleri Spor Bilimleri Dergisi, 15(2), 223–229. doi:10.5336/sportsci.2022-94239.

MAN-04. Bilen, E., & Elioz, M. (2023). The relationship between the SLC6A4 gene polymorphism (rs 5-HTTLPR) and aggression in combat athletes. Journal of ROL Sport Sciences, 4(4), 1423–1436. doi:10.5281/zenodo.10377634.

MAN-05. Yılmaz, Ö. Ö., Polat, T., Tacal Aslan, B., Yüksel, İ., Göçücü, K., Zileli, R., & Ulucan, K. (2024). Determination of COL1A1 rs1800012 polymorphism related to ligament and tendon injury in Turkish professional bodybuilders: Experimental research. Türkiye Klinikleri Spor Bilimleri Dergisi, 16(1), 1–6. doi:10.5336/sportsci.2023-97720.

MAN-06. Erkan, E., Eken, B. F., Kazancı, D., Polat, T., Yılmaz, Ö. Ö., Doğan, C. S., Tacal Aslan, B., Akkoç, O., & Ulucan, K. (2022). Türk uzun ve kısa mesafe koşucularında anjiotensinojen geni rs699 polimorfizminin atletik performans üzerine etkisi. Eurasian Research in Sport Science, 7(1), 1–8. doi:10.29228/ERISS.17.

MAN-07. Eken, B. F., Şahin, Z. N., Llewellyn-Waters, K., Tuna, G., Saç, A., Oktay, N. Ş., & Ulucan, K. (2023). Investigation of vitamin D receptor gene rs731236 polymorphism in Turkish rugby players. Eurasian Research in Sport Science, 8(1), 33–40. doi:10.29228/ERISS.28.

MAN-08. Duran, K., & Eliöz, M. (2024). SLC6A4 gene 5-HTTLPR distribution in futsal and football players. Journal of ROL Sport Sciences, 5(1), 112–124. doi:10.5281/zenodo.10886110.

MAN-09. Bulğay, C., Kasakolu, A., Kazan, H. H., Koncagul, S., Ergun, M. A., & Cerit, M. (2025). Examination of the relationship between angiotensin converting enzyme (ACE) rs4343 gene polymorphism and Yo-Yo intermittent recovery test (level 2) performance in non-elite athletes. Journal of Physical Education and Sport Health, 25(1), 1123–1130. doi:10.46733/PESH25141123d.

Y1088. Unver S, Atan T, Nursal AF, Yigit S. Association between NOS3 intron 4 VNTR variant and sports performance. Ann Clin Anal Med. 2022;13(2):225–229. doi:10.4328/ACAM.20987.

Y1089. Eken BF, Savci AA, Polat T, Yilmaz OO, Silar C, Tacal Aslan B, Oktay S, Ulucan K. Preliminary findings of the distribution of alpha-2-adrenergic receptor ADRA2A rs1800544 polymorphisms in kickboxing players. Clin Exp Health Sci. 2021;11(4):742–745. doi:10.33808/clinexphealthsci.863591.
