# Supplementary material for: Sports genomics research in Turkish athlete populations 2019 to 2026: a systematic review
Source: Front Genet. 2026 Oct 23;17:1921682. doi: 10.3389/fgene.2026.1921682 (PMC13739473; doi:10.3389/fgene.2026.1921682)
Supplement: Supplementary file 2 [file DataSheet2.pdf]

Supplementary Table S2 Search Strategies and Database Records

Full database-specific search strategies used in this systematic review. Search coverage was 1 January 2019 to 1 April 2026. Google Scholar was used as a supplementary source. Because its interface, ranking, and reported result counts are dynamic and not fully reproducible, the first 200 results returned by the systematic search were captured and screened (Bramer, 2016). This prespecified limit was consistent with a published recommendation to focus title screening on the first 200–300 Google Scholar results in systematic-review searching (Haddaway et al., 2015). Embase was not searched because institutional access was unavailable.

Database	Exact strategy or implementation	Date limit	Records
Web of Science Core Collection	TS=(("sport" OR "athlete" OR "athletic performance" OR "spor") AND ("genetic" OR "genetik") AND ("polymorphism" OR "genotype" OR "polimorfizm"))	01 Jan 2019–01 Apr 2026	324
Scopus	TITLE-ABS-KEY(("sport" OR "athlete" OR "athletic performance" OR "spor") AND ("genetic" OR "genetik") AND ("polymorphism" OR "genotype" OR "polimorfizm"))	01 Jan 2019–01 Apr 2026	882
PubMed/MEDLINE	("sport"[Title/Abstract] OR "athlete"[Title/Abstract] OR "athletic performance"[Title/Abstract] OR "spor"[Title/Abstract]) AND ("genetic"[Title/Abstract] OR "genetik"[Title/Abstract]) AND ("polymorphism"[Title/Abstract] OR "genotype"[Title/Abstract] OR "polimorfizm"[Title/Abstract])	2019/01/01–2026/04/01	225
SPORTDiscus with Full Text via EBSCOhost	(TI sport-block OR AB sport-block OR SU sport-block) AND (TI genetic-block OR AB genetic-block OR SU genetic-block) AND (TI polymorphism-block OR AB polymorphism-block OR SU polymorphism-block); each block used sport/athlete/athletic performance/spor; genetic/genetik; and polymorphism/genotype/polimorfizm terms respectively. AI Search off; no Full Text or Peer Reviewed limit.	01/2019–04/2026	84
Google Scholar	("sport" OR "athlete" OR "athletic performance" OR "spor") AND ("genetic" OR "genetik") AND ("polymorphism" OR "genotype" OR "polimorfizm"). Google Scholar was used as a supplementary source. Because the interface and reported result counts are dynamic and not fully reproducible, the first 200 results were systematically captured and screened (Bramer, 2016). This limit was consistent with the published recommendation to focus title screening on the first 200–300 Google Scholar results (Haddaway et al., 2015).	01 Jan 2019–01 Apr 2026	200
TRDizin	Searches were conducted primarily in the abstract field using smaller Turkish and English keyword combinations because the platform did not reliably execute the full Boolean strategy. Sport block: "spor", "sport", "athlete", and "athletic performance". Genetics block: "genetik" and "genetic". Genetic-variation block: "polimorfizm", "polymorphism", and "genotype". Records were date checked and deduplicated.	01 Jan 2019–01 Apr 2026	5

Database	Exact strategy or implementation	Date limit	Records
DergiPark	Twelve simpler searches combined three Turkish/English keyword blocks because the platform did not reliably execute the full Boolean strategy. Sport/population block: "sport", "spor", "athlete", and "athletic performance". Genetics block: "genetic" and "genetik". Genetic-variation block: "polymorphism", "polimorfizm", and "genotype". The 12 searches yielded 67 record views; deduplication by DergiPark record number yielded 21 unique records, and one post-cutoff 2026 record was removed.	01 Jan 2019–01 Apr 2026	20

Note. Identification total before deduplication: 1,740 records. The 2026 records in Web of Science and Scopus were individually audited against the 1 April 2026 cutoff. The search terms and combinations are listed in the table exactly as they were used during the search (in both Turkish and English).

Bramer WM. Variation in number of hits for complex searches in Google Scholar. Journal of the Medical Library Association. 2016;104(2):143–145. doi:10.3163/1536-5050.104.2.009.

Haddaway NR, Collins AM, Coughlin D, Kirk S. The role of Google Scholar in evidence reviews and its applicability to grey literature searching. PLoS One. 2015;10(9):e0138237. doi:10.1371/journal.pone.0138237.
