# Supplementary material for: Sports genomics research in Turkish athlete populations 2019 to 2026: a systematic review
Source: Front Genet. 2026 Oct 23;17:1921682. doi: 10.3389/fgene.2026.1921682 (PMC13739473; doi:10.3389/fgene.2026.1921682)
Supplement: Supplementary file 3 [file DataSheet3.pdf]

Supplementary Table S3 Full Text Eligibility Assessment

All 396 database-derived reports assessed in full text. Eligibility was assessed against the prespecified population-based criteria. "Included" denotes database-derived inclusion; nine additional reports identified through backward reference-list checking and forward citation searching are documented in the report-level dataset and PRISMA audit.

Report ID	Title	Decision	Code	Reason for decision
Y0002	The Relationship between Changes in MYBPC3 Single-Nucleotide Polymorphism-Associated Metabolites and Elite Athletes' Adaptive Cardiac Function.	Exclude	D8	The publication type was ineligible because it did not report original primary human data.
Y0004	Effect of Physical Activity on Chronic Widespread Pain: Insights From Meta-Analysis and Two-Sample Mendelian Randomization	Exclude	D8	The publication type was ineligible because it did not report original primary human data.
Y0005	Bone mineral density in high-level endurance runners: Part B-genotype-dependent characteristics.	Exclude	D2	No identifiable Turkish athlete population or separately extractable Turkish athlete subgroup was available.
Y0008	Association between polymorphism rs6295 of HTR1A serotonin receptor gene and personality traits among athletes of combat sport.	Exclude	D2	No identifiable Turkish athlete population or separately extractable Turkish athlete subgroup was available.
Y0016	Effects of Exercise Addiction and the COL1A1 Gene rs1800012 Polymorphism on Injury Susceptibility in Elite Female Volleyball Players	Include		Original human research included an identifiable Turkish athlete population or separately extractable Turkish athlete subgroup, eligible inherited genetic or genomic data, and a sport-related phenotype.
Y0020	Examining Health Outcomes in Juvenile Idiopathic Arthritis: A Genetic Epidemiology Study	Exclude	D4	The study did not include an eligible athlete population.
Y0024	The impact of creatine levels on musculoskeletal health in the elderly: a mendelian randomization analysis	Exclude	D4	The study did not include an eligible athlete population.
Y0025	Association of Physical Activity With Neurofilament Light Chain Trajectories in Autosomal Dominant Frontotemporal Lobar Degeneration Variant Carriers	Exclude	D4	The study did not include an eligible athlete population.

Report ID	Title	Decision	Code	Reason for decision
Y0028	Investigation of the putative functional relevance of the IL-6 3'UTR genetic variants with athletic phenotype in Turkish triathletes	Include		Original human research included an identifiable Turkish athlete population or separately extractable Turkish athlete subgroup, eligible inherited genetic or genomic data, and a sport-related phenotype.
Y0033	Association study of the Taq1D rs1800498 polymorphism of the DRD2 gene with personality traits in a group of athletes	Exclude	D2	No identifiable Turkish athlete population or separately extractable Turkish athlete subgroup was available.
Y0035	Study of thrombophilia genetic polymorphisms and hemostasis parameters in a group of young athletes	Exclude	D2	No identifiable Turkish athlete population or separately extractable Turkish athlete subgroup was available.
Y0036	Identification of Genomic Predictors of Muscle Fiber Size.	Exclude	D2	No identifiable Turkish athlete population or separately extractable Turkish athlete subgroup was available.
Y0037	A whole genome sequencing approach to anterior cruciate ligament rupture–a twin study in two unrelated families	Exclude	D2	No identifiable Turkish athlete population or separately extractable Turkish athlete subgroup was available.
Y0039	Genetic Profile in Genes Associated with Sports Injuries in Elite Endurance Athletes	Exclude	D2	No identifiable Turkish athlete population or separately extractable Turkish athlete subgroup was available.
Y0041	The Relationship among the Performance and the ACE I/D and ACTN3 R577X Polymorphisms and some Anthropometric Factors in Arm Wrestlers.	Include		Original human research included an identifiable Turkish athlete population or separately extractable Turkish athlete subgroup, eligible inherited genetic or genomic data, and a sport-related phenotype.
Y0043	Speed and power-related gene polymorphisms associated with playing position in elite soccer players	Exclude	D2	No identifiable Turkish athlete population or separately extractable Turkish athlete subgroup was available.
Y0046	Dissecting the causal association between social or physical inactivity and depression: a bidirectional two-sample Mendelian Randomization study	Exclude	D4	The study did not include an eligible athlete population.
Y0052	Angiotensin-converting gene and hypoxic exercise tolerance: A randomized crossover trial	Exclude	D2	No identifiable Turkish athlete population or separately extractable Turkish athlete subgroup was available.
Y0054	The effect of caffeine on exercise performance is not influenced by ADORA2A genotypes, alone or pooled with CYP1A2 genotypes, in adolescent athletes	Exclude	D2	No identifiable Turkish athlete population or separately extractable Turkish athlete subgroup was available.

Report ID	Title	Decision	Code	Reason for decision
Y0057	Associations between leisure sedentary behaviors, physical activity, and chronic respiratory diseases: a Mendelian randomization study	Exclude	D4	The study did not include an eligible athlete population.
Y0059	Association analysis of the OPRM1 polymorphism gene and personality traits among a cohort of professional athletes	Exclude	D2	No identifiable Turkish athlete population or separately extractable Turkish athlete subgroup was available.
Y0060	Disease Progression in Exercise-Induced Arrhythmogenic Cardiomyopathy Compared With Arrhythmogenic Right Ventricular Cardiomyopathy.	Exclude	D2	No identifiable Turkish athlete population or separately extractable Turkish athlete subgroup was available.
Y0061	Heavy DIY Activities as a Potential Preventative for Stress Urinary Incontinence	Exclude	D4	The study did not include an eligible athlete population.
Y0063	The ARK2N (C18ORF25) Genetic Variant Is Associated with Muscle Fiber Size and Strength Athlete Status.	Include		Original human research included an identifiable Turkish athlete population or separately extractable Turkish athlete subgroup, eligible inherited genetic or genomic data, and a sport-related phenotype.
Y0075	Effects of the Trp64Arg Polymorphism in the ADRB3 Gene on Body Composition, Cardiorespiratory Fitness, and Physical Activity in Healthy Adults	Exclude	D4	The study did not include an eligible athlete population.
Y0076	Extravascular implantable cardioverter-defibrillator therapy in patients with genetic heart disease: An early single-center experience	Exclude	D4	The study did not include an eligible athlete population.
Y0077	Matrix Metalloproteinase Polymorphisms as Genetic Risk Factors for Anterior Cruciate Ligament Injuries in Football Players: A Case–Control Study	Exclude	D2	No identifiable Turkish athlete population or separately extractable Turkish athlete subgroup was available.
Y0079	A Genome-Wide Association Study Reveals Two Genetic Markers for Chondromalacia	Exclude	D4	The study did not include an eligible athlete population.
Y0081	Beyond the Comfort Zone: Strenuous Sports as a Preventive Tactic Against Benign Prostatic Hyperplasia	Exclude	D4	The study did not include an eligible athlete population.
Y0082	Effectiveness of blood steroidal passport markers for detecting testosterone abuse in Asians	Exclude	D2	No identifiable Turkish athlete population or separately extractable Turkish athlete subgroup was available.

Report ID	Title	Decision	Code	Reason for decision
Y0084	Genetic variants and anterior cruciate ligament rupture – Elastin proteins gene and fibromodulin gene polymorphisms	Exclude	D2	No identifiable Turkish athlete population or separately extractable Turkish athlete subgroup was available.
Y0087	Principal components analysis to evaluate complex association of polymorphisms in ACE and ACTN3 genes and the extent of cardiovascular adaptive changes in elite athletes.	Exclude	D2	No identifiable Turkish athlete population or separately extractable Turkish athlete subgroup was available.
Y0089	PPARA and IL6: exploring associations with athletic performance and genotype polymorphism.	Include		Original human research included an identifiable Turkish athlete population or separately extractable Turkish athlete subgroup, eligible inherited genetic or genomic data, and a sport-related phenotype.
Y0097	Association of the CKM rs8111989 Polymorphism with Injury Epidemiology in Football Players	Exclude	D2	No identifiable Turkish athlete population or separately extractable Turkish athlete subgroup was available.
Y0099	Association between strenuous sports or other exercises and lung cancer risk: a mendelian randomization study	Exclude	D4	The study did not include an eligible athlete population.
Y0100	Association between uncoupling protein 1-3826 A/G polymorphism and the effects of anaerobic exercises on serum oxidative stress; [Relation entre le polymorphisme de la protéine découplante 1-3826 A/G et les effets des exercices anaérobies sur le stress oxydatif sérique]	Include		Original human research included an identifiable Turkish athlete population or separately extractable Turkish athlete subgroup, eligible inherited genetic or genomic data, and a sport-related phenotype.
Y0103	Bradycardia in a young competitive athlete	Exclude	D10	The record was a case report and was therefore ineligible.
Y0105	Associations of Brain-Derived Neurotropic Factor rs6265 Gene Polymorphism with Personality Dimensions among Athletes	Exclude	D2	No identifiable Turkish athlete population or separately extractable Turkish athlete subgroup was available.
Y0110	Betaine supplementation modulates betaine concentration by methylenetetrahydrofolate reductase genotype, but has no effect on amino acid profile in healthy active males: A randomized placebo-controlled cross-over study	Exclude	D4	The study did not include an eligible athlete population.
Y0113	Outcomes and Burdens to Return-to-Play for Phenotype Negative Athletes With a Genetic Heart Disease.	Exclude	D2	No identifiable Turkish athlete population or separately extractable Turkish athlete subgroup was available.
Y0116	Prevalence of Polymorphism and Post-Training Expression of ACTN3 (R/X) and ACE (I/D) Genes in CrossFit Athletes	Exclude	D2	No identifiable Turkish athlete population or separately extractable Turkish athlete subgroup was available.

Report ID	Title	Decision	Code	Reason for decision
Y0120	The evaluation of superoxide dismutase 1 gene insertion/deletion variant in athletes	Include		Original human research included an identifiable Turkish athlete population or separately extractable Turkish athlete subgroup, eligible inherited genetic or genomic data, and a sport-related phenotype.
Y0127	Genome-Wide Association Study of Exercise Addiction Among Elite Wrestlers	Include		Original human research included an identifiable Turkish athlete population or separately extractable Turkish athlete subgroup, eligible inherited genetic or genomic data, and a sport-related phenotype.
Y0129	Association of Sleep Traits, Physical Activity, and Sedentary Leisure Behavior With Female Reproductive Health: A Two-Sample Mendelian Randomization Analysis	Exclude	D4	The study did not include an eligible athlete population.
Y0130	Multi-phase, multi-ethnic GWAS uncovers putative loci in predisposition to elite sprint and power performance, health and disease.	Exclude	D2	No identifiable Turkish athlete population or separately extractable Turkish athlete subgroup was available.
Y0131	Exploring the relationship between caffeine metabolism-related CYP1A2 rs762551 polymorphism and team sport athlete status and training adaptations	Include		Original human research included an identifiable Turkish athlete population or separately extractable Turkish athlete subgroup, eligible inherited genetic or genomic data, and a sport-related phenotype.
Y0134	Unraveling the Unsolved Mysteries of the Athletic Heart	Exclude	D8	The publication type was ineligible because it did not report original primary human data.
Y0146	Genotypic Influences on Actuators of Aerobic Performance in Tactical Athletes	Exclude	D2	No identifiable Turkish athlete population or separately extractable Turkish athlete subgroup was available.
Y0154	Are the Positions in the Word Ranking of Competitive Alpine Skiers Explainable by Prominent Polymorphisms in Regulatory Genes of Mechanical and Metabolic Muscle Functioning?	Exclude	D2	No identifiable Turkish athlete population or separately extractable Turkish athlete subgroup was available.
Y0156	Association of VDR Polymorphisms with Muscle Mass Development in Elite Young Soccer Players: A Pilot Study	Exclude	D2	No identifiable Turkish athlete population or separately extractable Turkish athlete subgroup was available.
Y0157	The PPARGC1A Gly482Ser polymorphism is associated with elite long-distance running performance.	Exclude	D2	No identifiable Turkish athlete population or separately extractable Turkish athlete subgroup was available.

Report ID	Title	Decision	Code	Reason for decision
Y0159	Genetic variation in TMEM106B alters microglial activation and cytokine responses in chronic traumatic encephalopathy	Exclude	D2	No identifiable Turkish athlete population or separately extractable Turkish athlete subgroup was available.
Y0161	Concussion-Associated Gene Variant COMT rs4680 Is Associated With Elite Rugby Athlete Status.	Exclude	D2	No identifiable Turkish athlete population or separately extractable Turkish athlete subgroup was available.
Y0162	CD36 polymorphism, sugary drinks, and sedentarism are associated with hypertriglyceridemic waist phenotype	Exclude	D4	The study did not include an eligible athlete population.
Y0163	Association of Gene Variants for Mechanical and Metabolic Muscle Quality with Cardiorespiratory and Muscular Variables Related to Performance in Skiing Athletes	Exclude	D2	No identifiable Turkish athlete population or separately extractable Turkish athlete subgroup was available.
Y0169	TAX1BP3 Causes TRPV4-Mediated Autosomal Recessive Arrhythmogenic Cardiomyopathy	Exclude	D4	The study did not include an eligible athlete population.
Y0172	Metformin attenuates symptoms of osteoarthritis: role of genetic diversity of Bcl2 and CXCL16 in OA.	Exclude	D4	The study did not include an eligible athlete population.
Y0175	Observations from a prospective small cohort study suggest that CGRP genes contribute to acute posttraumatic headache burden after concussion	Exclude	D2	No identifiable Turkish athlete population or separately extractable Turkish athlete subgroup was available.
Y0176	Association of DRD2 and BDNF Genetic Polymorphisms with Exercise Addiction	Exclude	D2	No identifiable Turkish athlete population or separately extractable Turkish athlete subgroup was available.
Y0177	Dopaminergic Modulation of Conscientiousness: DRD2 rs1799732 and Personality Traits in Elite Mixed Martial Arts Athletes.	Exclude	D2	No identifiable Turkish athlete population or separately extractable Turkish athlete subgroup was available.
Y0178	Variants within the MMP3 and COL5A1 genes associate with soft tissue injury history in elite male rugby athletes.	Exclude	D2	No identifiable Turkish athlete population or separately extractable Turkish athlete subgroup was available.
Y0181	Nutritional Intervention and Ergogenic Aids in Sport Performance and Recovery	Exclude	D8	The publication type was ineligible because it did not report original primary human data.
Y0183	Gene variants previously associated with reduced soft-tissue injury risk: Part 2 - Polygenic associations with elite status in Rugby.	Exclude	D2	No identifiable Turkish athlete population or separately extractable Turkish athlete subgroup was available.

Report ID	Title	Decision	Code	Reason for decision
Y0186	Association Between the rs4680 Polymorphism of the COMT Gene and Personality Traits among Combat Sports Athletes.	Exclude	D2	No identifiable Turkish athlete population or separately extractable Turkish athlete subgroup was available.
Y0188	Genetic causal relationship between physical activity and osteoarthritis: a two-sample Mendelian randomization study	Exclude	D4	The study did not include an eligible athlete population.
Y0191	The Relationship between Genetic Variability and Seasonal Changes in Vertical Jump Performance in Amateur Soccer Players	Exclude	D2	No identifiable Turkish athlete population or separately extractable Turkish athlete subgroup was available.
Y0192	Using deep learning to predict abdominal age from liver and pancreas magnetic resonance images	Exclude	D4	The study did not include an eligible athlete population.
Y0197	Contributing Factors to Sensorimotor Adaptability in Reactive Agility Performance in Youth Athletes.	Exclude	D2	No identifiable Turkish athlete population or separately extractable Turkish athlete subgroup was available.
Y0198	Cardiac desmosomal reserve: Another piece of the exercise-induced arrhythmogenic cardiomyopathy puzzle?	Exclude	D8	The publication type was ineligible because it did not report original primary human data.
Y0201	Genetic profiles to identify talents in elite endurance athletes and professional football players.	Exclude	D2	No identifiable Turkish athlete population or separately extractable Turkish athlete subgroup was available.
Y0203	Evaluation of a circadian rhythm gene (PER3) VNTR variant in Turkish athletes	Include		Original human research included an identifiable Turkish athlete population or separately extractable Turkish athlete subgroup, eligible inherited genetic or genomic data, and a sport-related phenotype.
Y0206	Association of Genetic Profile with Muscle Mass Gain and Muscle Injury Prevention in Professional Football Players after Creatine Supplementation	Exclude	D2	No identifiable Turkish athlete population or separately extractable Turkish athlete subgroup was available.
Y0207	Impact of the DRD2 Polymorphisms on the Effectiveness of the Training Program	Exclude	D4	The study did not include an eligible athlete population.
Y0209	Genetic biomarkers of skiers from the Oğlağo Tribe in Muş Province, Turkey: An analysis of ACTN3, VEGF-A, and GABPB1 polymorphisms.	Include		Original human research included an identifiable Turkish athlete population or separately extractable Turkish athlete subgroup, eligible inherited genetic or genomic data, and a sport-related phenotype.
Y0211	Prevalence of ACE I/D and ACTN3 R577X genetic polymorphisms in Taekwondo athletes.	Exclude	D2	No identifiable Turkish athlete population or separately extractable Turkish athlete subgroup was available.

Report ID	Title	Decision	Code	Reason for decision
Y0212	Sex Differences in the Association between Risk of Anterior Cruciate Ligament Rupture and COL5A1 Polymorphisms in Elite Footballers.	Exclude	D2	No identifiable Turkish athlete population or separately extractable Turkish athlete subgroup was available.
Y0213	Evaluation of the 18-bp deletion/insertion variant of the VEGF gene in athletes.	Include		Original human research included an identifiable Turkish athlete population or separately extractable Turkish athlete subgroup, eligible inherited genetic or genomic data, and a sport-related phenotype.
Y0214	Genetic polymorphisms of muscular fitness in young healthy men	Exclude	D2	No identifiable Turkish athlete population or separately extractable Turkish athlete subgroup was available.
Y0218	Nutritional Status and Ergogenic Aids in Performance During Exercise and Sports	Exclude	D8	The publication type was ineligible because it did not report original primary human data.
Y0219	Genome-Wide Association Study Identifies CDKN1A as a Novel Locus Associated with Muscle Fiber Composition.	Exclude	D2	No identifiable Turkish athlete population or separately extractable Turkish athlete subgroup was available.
Y0221	ACTN3 R577X variant: could it be a determinant of sports performance in elite athletes in a Turkish population? A. Canikli et al.	Include		Original human research included an identifiable Turkish athlete population or separately extractable Turkish athlete subgroup, eligible inherited genetic or genomic data, and a sport-related phenotype.
Y0223	Replacing Sedentary Behavior Time With Physical Activities, Recommended Physical Activity, and Incident Coronary Heart Disease	Exclude	D4	The study did not include an eligible athlete population.
Y0225	CLINICAL APPLICATION OF GENE MEDICINE IN PREVENTING SPORTS INJURIES; [APLICAÇÃO CLÍNICA DA MEDICINA GENÉTICA NA PREVENÇÃO DE LESÕES ESPORTIVAS]; [APLICACIÓN CLÍNICA DE LA MEDICINA GENÉTICA EN LA PREVENCIÓN DE LESIONES DEPORTIVAS]	Exclude	D2	No identifiable Turkish athlete population or separately extractable Turkish athlete subgroup was available.
Y0226	Association Between COL5a1, COL11a1, and COL11a2 Gene Variations and Rotator Cuff Tendinopathy in Young Athletes	Exclude	D2	No identifiable Turkish athlete population or separately extractable Turkish athlete subgroup was available.

Report ID	Title	Decision	Code	Reason for decision
Y0228	Integrating pharmacogenetics in sport medicine: Enhancing treatment precision and preventing unintentional doping violation	Exclude	D8	The publication type was ineligible because it did not report original primary human data.
Y0237	ASSOCIATION OF CANDIDATE GENES REGULATING PROTEOLYTIC SYSTEMS WITH RUNNING PERFORMANCES AT DIFFERENT DISTANCES; [АССОЦИАЦИЯ КАНДИДАТНЫХ ГЕНОВ, РЕГУЛИРУЮЩИХ ПРОТЕОЛИТИЧЕСКИЕ СИСТЕМЫ КРОВИ С ЭФФЕКТИВНОСТЬЮ БЕГА НА ИЗБРАННОЙ ДИСТАНЦИИ]	Exclude	D2	No identifiable Turkish athlete population or separately extractable Turkish athlete subgroup was available.
Y0238	The effect of the ACTN3 gene variation on physically active individuals' susceptibility to muscle injury	Exclude	D2	No identifiable Turkish athlete population or separately extractable Turkish athlete subgroup was available.
Y0239	Congenital central hypoventilation syndrome in children: a Hong Kong perspective	Exclude	D4	The study did not include an eligible athlete population.
Y0241	Gene variants previously associated with reduced soft tissue injury risk: Part 1 - independent associations with elite status in rugby.	Exclude	D2	No identifiable Turkish athlete population or separately extractable Turkish athlete subgroup was available.
Y0242	The Relationship between ACE, ACTN3 and MCT1 Genetic Polymorphisms and Athletic Performance in Elite Rugby Union Players: A Preliminary Study.	Exclude	D2	No identifiable Turkish athlete population or separately extractable Turkish athlete subgroup was available.
Y0256	Genetic basis of hemodynamic response to controlled physical activity assessment in the elderly	Exclude	D4	The study did not include an eligible athlete population.
Y0257	GWAS Analysis of Sudden Cardiac Death Cases in a Turkish Population	Exclude	D7	No eligible sport-related phenotype was reported.
Y0258	The Association between Total Genotype Score and Athletic Performance in Weightlifters.	Exclude	D2	No identifiable Turkish athlete population or separately extractable Turkish athlete subgroup was available.
Y0259	Interaction Between the VNTR of the DAT1 Gene and DAT1 Methylation in Relation to Impulsivity in Combat Sports Athletes.	Exclude	D2	No identifiable Turkish athlete population or separately extractable Turkish athlete subgroup was available.

Report ID	Title	Decision	Code	Reason for decision
Y0264	Comparison of potential biomarker, ACTN3 rs1815739 polymorphism, for athletic performance of Turkish athletes.	Include		Original human research included an identifiable Turkish athlete population or separately extractable Turkish athlete subgroup, eligible inherited genetic or genomic data, and a sport-related phenotype.
Y0267	Genetic Profiling and Performance Optimization in Elite Combat Sport Athletes: A Cross-Sectional Study Based on Total Genetic Score Analysis.	Exclude	D2	No identifiable Turkish athlete population or separately extractable Turkish athlete subgroup was available.
Y0268	SCN9A rs6746030 Polymorphism and Pain Perception in Combat Athletes and Non-Athletes	Exclude	D2	No identifiable Turkish athlete population or separately extractable Turkish athlete subgroup was available.
Y0271	Association between peroxisome proliferator activated receptor gamma coactivator 1 gene with overweight and obesity risk: Case–control study and meta-analysis	Exclude	D8	The publication type was ineligible because it did not report original primary human data.
Y0276	Moderate exercise reveals the influence of ACTN3 R577X and ACE I/D polymorphisms on physical performance in non-athlete active subjects	Exclude	D4	The study did not include an eligible athlete population.
Y0278	The role of body fat in multiple sclerosis susceptibility and severity: A Mendelian randomisation study	Exclude	D4	The study did not include an eligible athlete population.
Y0279	TP-fusion at peak exercise: a novel marker for the recognition of unsuspected long QT syndrome patients.	Exclude	D4	The study did not include an eligible athlete population.
Y0284	Evaluation of the Association of VDR rs2228570 Polymorphism with Elite Track and Field Athletes' Competitive Performance.	Include		Original human research included an identifiable Turkish athlete population or separately extractable Turkish athlete subgroup, eligible inherited genetic or genomic data, and a sport-related phenotype.
Y0285	Effect of ADORA2A Gene Polymorphism and Acute Caffeine Supplementation on Hormonal Response to Resistance Exercise: A Double-Blind, Crossover, Placebo-Controlled Study	Exclude	D2	No identifiable Turkish athlete population or separately extractable Turkish athlete subgroup was available.
Y0288	A catechol-O-methyltransferase genetic variant impacts functional movement in tactical athletes	Exclude	D2	No identifiable Turkish athlete population or separately extractable Turkish athlete subgroup was available.

Report ID	Title	Decision	Code	Reason for decision
Y0289	Correlations between sports-related polygenic profiles, postural stability, power and strength performances of elite football players.	Exclude	D2	No identifiable Turkish athlete population or separately extractable Turkish athlete subgroup was available.
Y0291	On the aspiration to decode the impact of genomics on performance in power and endurance sports	Exclude	D8	The publication type was ineligible because it did not report original primary human data.
Y0293	Influence of type I collagen polymorphisms and risk of anterior cruciate ligament rupture in athletes: a case-control study	Exclude	D2	No identifiable Turkish athlete population or separately extractable Turkish athlete subgroup was available.
Y0299	TTN Variants Are Associated with Physical Performance and Provide Potential Markers for Sport-Related Phenotypes	Exclude	D2	No identifiable Turkish athlete population or separately extractable Turkish athlete subgroup was available.
Y0301	The prevalence of IGF-I axis genetic polymorphisms among decathlon athletes.	Exclude	D2	No identifiable Turkish athlete population or separately extractable Turkish athlete subgroup was available.
Y0304	Investigating a Variant in the Vitamin D Receptor Gene in Ahvaz Male and Female Athletes Compared to the Control Group with Normal Body Mass	Exclude	D2	No identifiable Turkish athlete population or separately extractable Turkish athlete subgroup was available.
Y0305	Association between ACE, ACTN3, AGT, BDKRB2, and IL-6 gene polymorphisms and elite status in Colombian athletes.	Exclude	D2	No identifiable Turkish athlete population or separately extractable Turkish athlete subgroup was available.
Y0306	Association of the G>A (rs6265) polymorphism in the brain derived neurotrophic factor gene (BDNF) with post-training changes in Caucasian women	Exclude	D4	The study did not include an eligible athlete population.
Y0307	Association of the ACTN3 Gene's Single-Nucleotide Variant Rs1815739 (R577X) with Sports Qualification and Competitive Distance in Caucasian Athletes of the Southern Urals.	Exclude	D2	No identifiable Turkish athlete population or separately extractable Turkish athlete subgroup was available.
Y0310	Genetic Factors of Elite Wrestling Status: A Multi-Ethnic Comparative Study.	Include		Original human research included an identifiable Turkish athlete population or separately extractable Turkish athlete subgroup, eligible inherited genetic or genomic data, and a sport-related phenotype.
Y0311	ANALYSIS OF PHYSICAL PERFORMANCE OF FOOTBALL PLAYERS CONSIDERING POLYMORPHISM OF ENDURANCE GENES	Exclude	D2	No identifiable Turkish athlete population or separately extractable Turkish athlete subgroup was available.

Report ID	Title	Decision	Code	Reason for decision
Y0313	Genome-Wide Analysis of Exertional Rhabdomyolysis in Sickle Cell Trait Positive African Americans	Exclude	D4	The study did not include an eligible athlete population.
Y0317	Association of Alzheimer's Disease Polygenic Risk Score with Concussion Severity and Recovery Metrics	Exclude	D2	No identifiable Turkish athlete population or separately extractable Turkish athlete subgroup was available.
Y0318	Anterior cruciate ligament injury and its postoperative outcomes are not associated with polymorphism in COL1A1 rs1107946 (G/T): a case-control study in the Middle East elite athletes.	Exclude	D2	No identifiable Turkish athlete population or separately extractable Turkish athlete subgroup was available.
Y0320	EFFECTS OF GENETIC VARIATIONS OF MLCK2, AMPD1, AND COL5A1 ON MUSCLE ENDURANCE; [EFEITOS DAS VARIAÇÕES GENÉTICAS DE MLCK2, AMPD1 E COL5A1 SOBRE A RESISTÊNCIA MUSCULAR]; [EFECTOS DE LAS VARIACIONES GENÉTICAS DE MLCK2, AMPD1 Y COL5A1 SOBRE LA RESISTENCIA MUSCULAR]	Include		Original human research included an identifiable Turkish athlete population or separately extractable Turkish athlete subgroup, eligible inherited genetic or genomic data, and a sport-related phenotype.
Y0322	Allelic Frequency of Two Polymorphisms (rs1815739) and (rs2228570) in Alphaactinin3 and Vitamin D Absorption Receptor Genes in Athletes Population	Exclude	D2	No identifiable Turkish athlete population or separately extractable Turkish athlete subgroup was available.
Y0324	Concussion-Associated Polygenic Profiles of Elite Male Rugby Athletes.	Exclude	D2	No identifiable Turkish athlete population or separately extractable Turkish athlete subgroup was available.
Y0325	Different intensities of physical activity for amyotrophic lateral sclerosis and Parkinson disease: A Mendelian randomization study and meta-analysis	Exclude	D4	The study did not include an eligible athlete population.
Y0331	The ADORA2A TT Genotype Is Associated with Anti-Inflammatory Effects of Caffeine in Response to Resistance Exercise and Habitual Coffee Intake	Exclude	D2	No identifiable Turkish athlete population or separately extractable Turkish athlete subgroup was available.
Y0335	Influence of Genetic Polymorphisms and Biochemical Biomarkers on Response to Nutritional Iron Supplementation and Performance in a Professional Football Team: A Pilot Longitudinal Study.	Exclude	D2	No identifiable Turkish athlete population or separately extractable Turkish athlete subgroup was available.

Report ID	Title	Decision	Code	Reason for decision
Y0339	Association Between the COL5A1 rs12722 Genotype and the Prevalence of Anterior Cruciate Ligament Rupture in Professional Football Players.	Exclude	D2	No identifiable Turkish athlete population or separately extractable Turkish athlete subgroup was available.
Y0346	Assessment of fibrillin-2 and elastin gene polymorphisms in patients with a traumatic Achilles tendon rupture: Is Achilles tendon rupture a genetic disease?	Exclude	D2	No identifiable Turkish athlete population or separately extractable Turkish athlete subgroup was available.
Y0348	Genetic Markers that Predict the Formation of Certain Body Types and the Development of Motor Qualities	Exclude	D2	No identifiable Turkish athlete population or separately extractable Turkish athlete subgroup was available.
Y0349	Association of Gene Variants with Seasonal Variation in Muscle Strength and Aerobic Capacity in Elite Skiers	Exclude	D2	No identifiable Turkish athlete population or separately extractable Turkish athlete subgroup was available.
Y0358	Prevalence of Ten Gene Variants Involved in Muscular Phenotypes in a Mexican Mestizo Population.	Exclude	D4	The study did not include an eligible athlete population.
Y0359	Association analysis of indel variants and gene expression identifies <i>MDM4</i> as a novel locus for skeletal muscle hypertrophy and power athlete status	Include		Original human research included an identifiable Turkish athlete population or separately extractable Turkish athlete subgroup, eligible inherited genetic or genomic data, and a sport-related phenotype.
Y0360	Association of bradykinin receptor 2 (BDKRB2) variants with physical performance and muscle mass: Findings from the LACE sarcopenia trial	Exclude	D4	The study did not include an eligible athlete population.
Y0362	Distinguishing left ventricular hypertrophy from hypertrophic cardiomyopathy in adolescents: a longitudinal observation study	Exclude	D4	The study did not include an eligible athlete population.
Y0363	Genetic profile in genes associated with muscle injuries and injury etiology in professional soccer players	Exclude	D2	No identifiable Turkish athlete population or separately extractable Turkish athlete subgroup was available.
Y0364	Association of ANKK1 and DRD2 gene polymorphisms with exercise addiction among elite athletes	Include		Original human research included an identifiable Turkish athlete population or separately extractable Turkish athlete subgroup, eligible inherited genetic or genomic data, and a sport-related phenotype.
Y0368	Genetic association of social participation with cognitive function: A bidirectional Mendelian randomization study	Exclude	D4	The study did not include an eligible athlete population.

Report ID	Title	Decision	Code	Reason for decision
Y0371	Association of APOE Genotypes and Chronic Traumatic Encephalopathy	Exclude	D4	The study did not include an eligible athlete population.
Y0372	Evaluation of demographic, clinical, and aetiological data of patients admitted to cardiology clinics and diagnosed with left ventricular hypertrophy in Turkish population (LVH-TR)	Exclude	D4	The study did not include an eligible athlete population.
Y0382	Creating a Lifelong Passion for Exercise: The Way Forward	Exclude	D8	The publication type was ineligible because it did not report original primary human data.
Y0383	Correlation between ACTN3 gene Polymorphism and Anthropometric Measurements of the Swimmers with Their Performance	Exclude	D2	No identifiable Turkish athlete population or separately extractable Turkish athlete subgroup was available.
Y0385	Replacement of leisure-time sedentary behavior with various physical activities and the risk of dementia incidence and mortality: A prospective cohort study	Exclude	D4	The study did not include an eligible athlete population.
Y0387	Detection of Oral Testosterone Undecanoate Administration in UGT2B17 del/del and del/ins Individuals. Part II: Urinary Endogenous Steroid Sulfate Markers	Exclude	D4	The study did not include an eligible athlete population.
Y0390	Effect of MCT1 A1470T Polymorphism on Lactate and Potassium Concentrations After Caffeine Ingestion During Acute Resistance Exercise	Exclude	D2	No identifiable Turkish athlete population or separately extractable Turkish athlete subgroup was available.
Y0398	The Genetic Basis of Decathlon Performance: An Exploratory Study	Exclude	D2	No identifiable Turkish athlete population or separately extractable Turkish athlete subgroup was available.
Y0399	Does the TMPRSS6 C > T Polymorphism Modify the Endurance Training Effects on Hematological Parameters?	Exclude	D4	The study did not include an eligible athlete population.
Y0400	Interactions between genetic risk for 21 neurodevelopmental and psychiatric disorders and sport activity on youth mental health	Exclude	D4	The study did not include an eligible athlete population.
Y0402	Association of ACTN3 R577X Polymorphism with Elite Basketball Player Status and Training Responses	Include		Original human research included an identifiable Turkish athlete population or separately extractable Turkish athlete subgroup, eligible inherited genetic or genomic data, and a sport-related phenotype.

Report ID	Title	Decision	Code	Reason for decision
Y0404	ACTN-3 and ECA genes expression do not influence the acute change in muscle mechanical and functional properties in youth handballers	Exclude	D2	No identifiable Turkish athlete population or separately extractable Turkish athlete subgroup was available.
Y0406	Causal Association Between Physical Activity, Leisure Sedentary Behaviors, Lifestyle Habits, and Multisite Chronic Pain: A Bidirectional Mendelian Randomization Study	Exclude	D4	The study did not include an eligible athlete population.
Y0409	Exploring the relationship between creatine supplementation and renal function: insights from Mendelian randomization analysis	Exclude	D4	The study did not include an eligible athlete population.
Y0414	The Distinguishing Factor in Soccer Players is Aerobic Performance, not the ACTN3 Gene; [El Factor Distintivo en los Jugadores de Fútbol es el Rendimiento Aeróbico y no el gen ACTN3]	Include		Original human research included an identifiable Turkish athlete population or separately extractable Turkish athlete subgroup, eligible inherited genetic or genomic data, and a sport-related phenotype.
Y0415	Community-Based Pilot Study of Chlamydia trachomatis and Neisseria gonorrhoeae Urogenital Infections Among Young Adults in the North and the Central Coast of Portugal	Exclude	D4	The study did not include an eligible athlete population.
Y0419	Genetically-predicted effects of lifestyle factors on frailty: Evidence from Mendelian randomization study	Exclude	D4	The study did not include an eligible athlete population.
Y0420	DRD2 Ex8 rs6276 Polymorphism and NEO-FFI Personality Traits in Elite Athletes and Controls.	Exclude	D2	No identifiable Turkish athlete population or separately extractable Turkish athlete subgroup was available.
Y0427	Genotype Distribution of the ACTN3 p.R577X Polymorphism in Elite Badminton Players: A Preliminary Study	Exclude	D2	No identifiable Turkish athlete population or separately extractable Turkish athlete subgroup was available.
Y0430	Gene Polymorphism and Total Genetic Score in Martial Arts Athletes with Different Athletic Qualifications	Exclude	D2	No identifiable Turkish athlete population or separately extractable Turkish athlete subgroup was available.
Y0431	The relationships between ACTN3 rs1815739 and PPARA-α rs4253778 gene polymorphisms and athletic performance characteristics in professional soccer players.	Exclude	D2	No identifiable Turkish athlete population or separately extractable Turkish athlete subgroup was available.
Y0436	Polymorphisms in Genes Encoding VDR, CALCR and Antioxidant Enzymes as Predictors of Bone Tissue Condition in Young, Healthy Men	Exclude	D4	The study did not include an eligible athlete population.

Report ID	Title	Decision	Code	Reason for decision
Y0437	The COL5A1 Gene Allelic Combination and ACL Injury Risk in Team Sport: A Preliminary Report; [Gene COL5A1 e o risco de lesões do LCA no esporte de equipe: Um relatório preliminar]	Exclude	D2	No identifiable Turkish athlete population or separately extractable Turkish athlete subgroup was available.
Y0440	Caffeine exacerbates exercise-induced gut cell damage and is influenced by ADORA2A genotype but not CYP1A2 genotype: A preliminary study	Exclude	D2	No identifiable Turkish athlete population or separately extractable Turkish athlete subgroup was available.
Y0442	Modifying effects of race and ethnicity and APOE on the association of physical activity with risk of Alzheimer's disease and related dementias	Exclude	D4	The study did not include an eligible athlete population.
Y0445	Atrial fibrillation in elite rowers: a genetic and endurance sport nexus?	Exclude	D8	The publication type was ineligible because it did not report original primary human data.
Y0449	Genomics and Biology of Exercise, Where Are We Now?	Exclude	D8	The publication type was ineligible because it did not report original primary human data.
Y0452	Mendelian randomization analysis reveals the impact of physical and occupational activities on the risk of gastroesophageal reflux disease and Barrett’s esophagus	Exclude	D4	The study did not include an eligible athlete population.
Y0454	Comparison of MCM6 rs4988235 Polymorphism Allele and Genotype Frequencies in Professional Football Players and a Sedentary Control Group	Include		Original human research included an identifiable Turkish athlete population or separately extractable Turkish athlete subgroup, eligible inherited genetic or genomic data, and a sport-related phenotype.
Y0457	Genoset for CALCA and RAMP1 Single Nucleotide Polymorphisms Are Related to the Magnitude of Headache Symptoms After Concussion: A Preliminary Observation	Exclude	D2	No identifiable Turkish athlete population or separately extractable Turkish athlete subgroup was available.
Y0459	Cumulative UV Exposure or a Modified SCINEXA™-Skin Aging Score Do Not Play a Substantial Role in Predicting the Risk of Developing Keratinocyte Cancers after Solid Organ Transplantation—A Case Control Study	Exclude	D4	The study did not include an eligible athlete population.

Report ID	Title	Decision	Code	Reason for decision
Y0464	Exome-Wide Association Study of Competitive Performance in Elite Athletes.	Include		Original human research included an identifiable Turkish athlete population or separately extractable Turkish athlete subgroup, eligible inherited genetic or genomic data, and a sport-related phenotype.
Y0468	The ACTN3 R577X Nonsense Allele Is Underrepresented in Professional Volleyball Players and Associated with an Increased Risk of Muscle Injury in Female Players	Include		Original human research included an identifiable Turkish athlete population or separately extractable Turkish athlete subgroup, eligible inherited genetic or genomic data, and a sport-related phenotype.
Y0470	Vigorous Exercise in Patients with Hypertrophic Cardiomyopathy	Exclude	D4	The study did not include an eligible athlete population.
Y0474	Genome-wide association study identifying variants related to performance and injury in high-performance athletes	Exclude	D2	No identifiable Turkish athlete population or separately extractable Turkish athlete subgroup was available.
Y0475	Changes in Vertical Jump Parameters After Training Unit in Relation to ACE, ACTN3, PPARA, HIF1A, and AMPD1 Gene Polymorphisms in Volleyball and Basketball Players	Exclude	D2	No identifiable Turkish athlete population or separately extractable Turkish athlete subgroup was available.
Y0480	Diagnosis and management of children with McArdle Syndrome (GSD V) in New South Wales	Exclude	D4	The study did not include an eligible athlete population.
Y0482	Sprint and Anaerobic Power with the Soccer-Specific ACTN3 Gene: A Distintive Example; [Sprint y Potencia Anaeróbica con el Gen ACTN3 Específico del Fútbol: Un Ejemplo Distintivo]	Include		Original human research included an identifiable Turkish athlete population or separately extractable Turkish athlete subgroup, eligible inherited genetic or genomic data, and a sport-related phenotype.
Y0483	Genetic correlation between female infertility and mental health and lifestyle factors: A linkage disequilibrium score regression study	Exclude	D4	The study did not include an eligible athlete population.
Y0486	The AMPD1 Gene’s rs17602729 Polymorphism and Athletic Performance in Track and Field Athletes	Include		Original human research included an identifiable Turkish athlete population or separately extractable Turkish athlete subgroup, eligible inherited genetic or genomic data, and a sport-related phenotype.
Y0487	Association of Genetically Predicted BCAA Levels with Muscle Fiber Size in Athletes Consuming Protein	Exclude	D2	No identifiable Turkish athlete population or separately extractable Turkish athlete subgroup was available.

Report ID	Title	Decision	Code	Reason for decision
Y0490	A pilot study examining BDNF Val66Met polymorphism and biological sex: Relationships with baseline cognitive functioning in adolescent athletes	Exclude	D2	No identifiable Turkish athlete population or separately extractable Turkish athlete subgroup was available.
Y0495	Description of a Cohort with a New Truncating MYBPC3 Variant for Hypertrophic Cardiomyopathy in Northern Spain.	Exclude	D4	The study did not include an eligible athlete population.
Y0496	Common genetic basis of ALS patients and soccer players may contribute to disease risk	Exclude	D2	No identifiable Turkish athlete population or separately extractable Turkish athlete subgroup was available.
Y0498	Association analysis of the dopaminergic receptor 2 gene Tag1B rs1079597 and personality traits among a cohort of professional athletes.	Exclude	D2	No identifiable Turkish athlete population or separately extractable Turkish athlete subgroup was available.
Y0500	Effects of Algae-Based Supplementation on Metabolic, Oxidative, and Inflammatory Markers in Physically Active Adults: A Pilot Randomized Controlled Trial	Exclude	D2	No identifiable Turkish athlete population or separately extractable Turkish athlete subgroup was available.
Y0507	Association of the Val158Met Polymorphism of the COMT Gene with Measures of Psychophysiological Status in Athletes	Exclude	D2	No identifiable Turkish athlete population or separately extractable Turkish athlete subgroup was available.
Y0510	Genome-Wide Association Study Reveals a Novel Association Between MYBPC3 Gene Polymorphism, Endurance Athlete Status, Aerobic Capacity and Steroid Metabolism.	Exclude	D2	No identifiable Turkish athlete population or separately extractable Turkish athlete subgroup was available.
Y0511	The relationship between ACTN3 R577X gene polymorphism and physical performance in amateur soccer players and sedentary individuals.	Include		Original human research included an identifiable Turkish athlete population or separately extractable Turkish athlete subgroup, eligible inherited genetic or genomic data, and a sport-related phenotype.
Y0512	Distribution of α-actinin-3 rs1815739 and angiotensin-1 converting enzyme InDel polymorphisms in Turkish bodybuilders	Include		Original human research included an identifiable Turkish athlete population or separately extractable Turkish athlete subgroup, eligible inherited genetic or genomic data, and a sport-related phenotype.
Y0516	NDUFB6 Polymorphism Is Associated With Physical Activity-Mediated Metabolic Changes in Type 2 Diabetes	Exclude	D4	The study did not include an eligible athlete population.

Report ID	Title	Decision	Code	Reason for decision
Y0517	Genetic influence in exercise performance	Exclude	D8	The publication type was ineligible because it did not report original primary human data.
Y0519	Caffeine, genetic variation and anaerobic performance in male athletes: a randomized controlled trial	Exclude	D2	No identifiable Turkish athlete population or separately extractable Turkish athlete subgroup was available.
Y0523	Genetic variants and mixed sport disciplines: A comparison among soccer, combat and motorcycle athletes	Exclude	D2	No identifiable Turkish athlete population or separately extractable Turkish athlete subgroup was available.
Y0524	Association of TNF-α -308G > A polymorphism with susceptibility to tendinopathy in athletes: a case–control study	Exclude	D2	No identifiable Turkish athlete population or separately extractable Turkish athlete subgroup was available.
Y0525	Familial arrhythmogenic cardiomyopathy: Clinical determinants of phenotype discordance and the impact of endurance sports	Exclude	D2	No identifiable Turkish athlete population or separately extractable Turkish athlete subgroup was available.
Y0527	Association analysis of polymorphic variants of the bdnf gene in athletes	Exclude	D2	No identifiable Turkish athlete population or separately extractable Turkish athlete subgroup was available.
Y0535	ACTN3 R577X Genotype in Professional and Amateur Tennis Players	Exclude	D2	No identifiable Turkish athlete population or separately extractable Turkish athlete subgroup was available.
Y0541	Caffeine improves various aspects of athletic performance in adolescents independent of their 163 C > A CYP1A2 genotypes.	Exclude	D2	No identifiable Turkish athlete population or separately extractable Turkish athlete subgroup was available.
Y0547	Study of Association of gene polymorphism with nutrition and nutritional status of martial arts athletes; [Изучение ассоциации полиморфизма генов с питанием и пищевым статусом спортсменов-единоборцев]	Exclude	D2	No identifiable Turkish athlete population or separately extractable Turkish athlete subgroup was available.
Y0549	The combined frequencies of the IL-6 G-174C and IGFBP3 A-202C polymorphisms among swimmers and runners	Exclude	D2	No identifiable Turkish athlete population or separately extractable Turkish athlete subgroup was available.
Y0551	EFFECT OF ACTN3 POLYMORPHISM ON SELF-REPORTED RUNNING TIMES.	Exclude	D2	No identifiable Turkish athlete population or separately extractable Turkish athlete subgroup was available.
Y0553	Genetic Polymorphisms Related to VO₂max Adaptation Are Associated With Elite Rugby Union Status and Competitive Marathon Performance.	Exclude	D2	No identifiable Turkish athlete population or separately extractable Turkish athlete subgroup was available.

Report ID	Title	Decision	Code	Reason for decision
Y0554	Genomic analysis reveals association of specific SNPs with athletic performance and susceptibility to injuries in professional soccer players.	Exclude	D2	No identifiable Turkish athlete population or separately extractable Turkish athlete subgroup was available.
Y0560	The effects of regular aerobic exercise training on blood nitric oxide levels and oxidized LDL and the role of eNOS intron 4a/b polymorphism	Exclude	D4	The study did not include an eligible athlete population.
Y0561	Excess heritability contribution of alcohol consumption variants in the "missing heritability" of type 2 diabetes mellitus	Exclude	D4	The study did not include an eligible athlete population.
Y0562	The Short Tandem Repeat of the DMT1 Gene as a Molecular Marker of Elite Long-Distance Runners.	Exclude	D2	No identifiable Turkish athlete population or separately extractable Turkish athlete subgroup was available.
Y0563	Transcranial Alternating Current Stimulation (tACS) Does Not Affect Sports People's Explosive Power: A Pilot Study.	Exclude	D2	No identifiable Turkish athlete population or separately extractable Turkish athlete subgroup was available.
Y0571	CYP1A2 Genotype Modifies the Effects of Caffeine Compared With Placebo on Muscle Strength in Competitive Male Athletes.	Exclude	D2	No identifiable Turkish athlete population or separately extractable Turkish athlete subgroup was available.
Y0574	High frequency of paternal iso or heterodisomy at chromosome 20 associated with sporadic pseudohypoparathyroidism 1B	Exclude	D4	The study did not include an eligible athlete population.
Y0577	Maladaptive coping strategies and neuroticism mediate the relationship between 5HTT-LPR polymorphisms and symptoms of anxiety in elite athletes	Exclude	D2	No identifiable Turkish athlete population or separately extractable Turkish athlete subgroup was available.
Y0581	Targeted genotype analyses of GWAS-derived lean body mass and handgrip strength-associated single-nucleotide polymorphisms in elite master athletes	Exclude	D2	No identifiable Turkish athlete population or separately extractable Turkish athlete subgroup was available.
Y0582	ACTN3 's R577X Single Nucleotide Polymorphism Allele Distribution Differs Significantly in Professional Football Players according to Their Field Position	Exclude	D2	No identifiable Turkish athlete population or separately extractable Turkish athlete subgroup was available.
Y0586	Genetic variation, inflammatory and muscle injury response in rugby players to different positions in the field	Exclude	D2	No identifiable Turkish athlete population or separately extractable Turkish athlete subgroup was available.

Report ID	Title	Decision	Code	Reason for decision
Y0587	Genomic prediction of tendinopathy risk in elite team sports	Exclude	D2	No identifiable Turkish athlete population or separately extractable Turkish athlete subgroup was available.
Y0593	The influence of the ACTN3 R577X polymorphism in the responsiveness to post-activation jump performance enhancement in untrained young men; [A influência do polimorfismo R577X do gene ACTN3 na responsividade à melhora de desempenho de salto pós-ativação em indivíduos jovens destreinados]	Exclude	D4	The study did not include an eligible athlete population.
Y0594	Interaction of APOE4 alleles and PET tau imaging in former contact sport athletes	Exclude	D2	No identifiable Turkish athlete population or separately extractable Turkish athlete subgroup was available.
Y0598	Social Isolation, Social Interaction, and Alzheimer's Disease: A Mendelian Randomization Study	Exclude	D4	The study did not include an eligible athlete population.
Y0599	Association between (GT)n Promoter Polymorphism and Recovery from Concussion: A Pilot Study	Exclude	D2	No identifiable Turkish athlete population or separately extractable Turkish athlete subgroup was available.
Y0600	Adiponectin gene rs1501299 polymorphism is associated with increased risk of anterior cruciate ligament rupture.	Exclude	D2	No identifiable Turkish athlete population or separately extractable Turkish athlete subgroup was available.
Y0601	Three genes associated with anterior and posterior cruciate ligament injury A GENOME-WIDE ASSOCIATION ANALYSIS	Exclude	D2	No identifiable Turkish athlete population or separately extractable Turkish athlete subgroup was available.
Y0602	Are MMP3, MMP8 and TIMP2 gene variants associated with anterior cruciate ligament rupture susceptibility?	Exclude	D2	No identifiable Turkish athlete population or separately extractable Turkish athlete subgroup was available.
Y0603	Novel associations between interleukin-15 polymorphisms and post-training changes of body composition parameters in young nonobese women	Exclude	D4	The study did not include an eligible athlete population.
Y0607	Codeine and Tramadol Use in Athletes: A Potential for Abuse	Exclude	D8	The publication type was ineligible because it did not report original primary human data.
Y0611	Association between pgc-1α gene variations and physical performance in non-athletic boys	Exclude	D4	The study did not include an eligible athlete population.

Report ID	Title	Decision	Code	Reason for decision
Y0612	A Case-Control Study Investigating the Effect of MTHFR C677T Variant on Performance of Elite Athletes.	Include		Original human research included an identifiable Turkish athlete population or separately extractable Turkish athlete subgroup, eligible inherited genetic or genomic data, and a sport-related phenotype.
Y0613	Exercise is Associated With Impaired Left Ventricular Systolic Function in Patients With Lamin A/C Genotype	Exclude	D4	The study did not include an eligible athlete population.
Y0619	Evaluation of the Association of COMT Rs4680 Polymorphism with Swimmers' Competitive Performance.	Exclude	D2	No identifiable Turkish athlete population or separately extractable Turkish athlete subgroup was available.
Y0620	The nos3-786 t/c polymorphism is associated with power performance in adolescent male basketball players	Exclude	D2	No identifiable Turkish athlete population or separately extractable Turkish athlete subgroup was available.
Y0623	Metabolic GWAS of elite athletes reveals novel genetically-influenced metabolites associated with athletic performance	Exclude	D2	No identifiable Turkish athlete population or separately extractable Turkish athlete subgroup was available.
Y0627	Harm Avoidance as a possible mediator in the relationship between the 5-HTTLPR and Cognitive Anxiety in High Level Athletes	Exclude	D2	No identifiable Turkish athlete population or separately extractable Turkish athlete subgroup was available.
Y0630	Effect of the brain-derived neurotrophic factor gene Val66Met polymorphism on sensory-motor integration during a complex motor learning exercise	Exclude	D4	The study did not include an eligible athlete population.
Y0632	A Genome-Wide Association Study of Sprint Performance in Elite Youth Football Players.	Exclude	D2	No identifiable Turkish athlete population or separately extractable Turkish athlete subgroup was available.
Y0633	Re-evaluation of combined ((ES/EG)/(TS/TG)) ratio as a marker of testosterone intake in men	Exclude	D4	The study did not include an eligible athlete population.
Y0637	No Change – No Gain; The Effect of Age, Sex, Selected Genes and Training on Physiological and Performance Adaptations in Cross-Country Skiing	Exclude	D2	No identifiable Turkish athlete population or separately extractable Turkish athlete subgroup was available.

Report ID	Title	Decision	Code	Reason for decision
Y0638	The study of Association of gene polymorphism with sports success and risk of alimentary-dependent diseases in athletes representing cyclic sports; [Изучение ассоциации полиморфизма генов со спортивной успешностью и риском развития алиментарно-зависимых заболеваний у спортсменов, представляющих циклические виды спорта]	Exclude	D2	No identifiable Turkish athlete population or separately extractable Turkish athlete subgroup was available.
Y0640	Physical activity, sedentary behavior and risk of coronary artery disease, myocardial infarction and ischemic stroke: a two-sample Mendelian randomization study	Exclude	D4	The study did not include an eligible athlete population.
Y0646	Somatotype and polymorphism of the ACTN3 and ACE gene in Chilean table tennis players; [Somatotipo y polimorfismo del gen ACTN3 y ECA en jugadores de tenis de mesa chilenos]	Exclude	D2	No identifiable Turkish athlete population or separately extractable Turkish athlete subgroup was available.
Y0648	Lack of association between the GNB3 rs5443, HIF1A rs11549465 polymorphisms, physiological and functional characteristics	Exclude	D2	No identifiable Turkish athlete population or separately extractable Turkish athlete subgroup was available.
Y0649	Peroxisome Proliferator-Activated Receptor Alpha (PPARα) rs4253778 Polymorphism in a Turkish Soccer Player Cohort	Include		Original human research included an identifiable Turkish athlete population or separately extractable Turkish athlete subgroup, eligible inherited genetic or genomic data, and a sport-related phenotype.
Y0651	GENETIC VARIABILITY AMONG POWER ATHLETES: THE STRONGER VS. THE FASTER.	Exclude	D2	No identifiable Turkish athlete population or separately extractable Turkish athlete subgroup was available.
Y0652	Single nucleotide polymorphism A-511 G of IL-1 gene modifies anthropometric and physiological parameters of athletes	Exclude	D2	No identifiable Turkish athlete population or separately extractable Turkish athlete subgroup was available.
Y0653	Analysis of Polymorphic Variants of the Dopamine Transporter (DAT1) Gene Polymorphism and Personality Traits Among Athletes.	Exclude	D2	No identifiable Turkish athlete population or separately extractable Turkish athlete subgroup was available.
Y0658	Toxic tau: The TAU gene polymorphisms associate with concussion history in rugby union players	Exclude	D2	No identifiable Turkish athlete population or separately extractable Turkish athlete subgroup was available.

Report ID	Title	Decision	Code	Reason for decision
Y0663	CYP1A2 genotype and acute effects of caffeine on resistance exercise, jumping, and sprinting performance	Exclude	D2	No identifiable Turkish athlete population or separately extractable Turkish athlete subgroup was available.
Y0666	Analysis of selected variants of drd2 and ankk1 genes in combat athletes	Exclude	D2	No identifiable Turkish athlete population or separately extractable Turkish athlete subgroup was available.
Y0667	Inferolateral T wave inversion in athletes: phenotype-genotype correlation	Exclude	D2	No identifiable Turkish athlete population or separately extractable Turkish athlete subgroup was available.
Y0668	Associations between the occurrence of the ACTN3 R577X, ACE I/D, BDKRB2 +9/-9 polymorphisms and anaerobic performance among a group of elite fencers	Exclude	D2	No identifiable Turkish athlete population or separately extractable Turkish athlete subgroup was available.
Y0669	The genetic profile of elite youth soccer players and its association with power and speed depends on maturity status.	Exclude	D2	No identifiable Turkish athlete population or separately extractable Turkish athlete subgroup was available.
Y0673	Association analysis of gene polymorphisms col1a, mct1, col12a1 with sports hernia in football players	Exclude	D2	No identifiable Turkish athlete population or separately extractable Turkish athlete subgroup was available.
Y0681	The electrocardiogram and the phenotypic expression of hypertrophic cardiomyopathy	Exclude	D8	The publication type was ineligible because it did not report original primary human data.
Y0684	World-class athletic performance and genetic endowment	Exclude	D8	The publication type was ineligible because it did not report original primary human data.
Y0687	Influence of genetic factors in elbow tendon pathology: a case-control study	Exclude	D2	No identifiable Turkish athlete population or separately extractable Turkish athlete subgroup was available.
Y0694	Analysis of the Frequency of Genetic Polymorphisms of Sports Athletes in the Republic of Belarus	Exclude	D2	No identifiable Turkish athlete population or separately extractable Turkish athlete subgroup was available.
Y0696	Predictors of performance in Amateur Olympic distance triathlon: Predictors in amateur triathlon	Exclude	D2	No identifiable Turkish athlete population or separately extractable Turkish athlete subgroup was available.
Y0956	ELİT TÜRK GÜREŞÇİLERİNİN ANJİOTENSİN I-DÖNÜŞTÜRÜCÜ ENZİM GEN POLİMORFİZMİ VE VO_{2MAX} DÜZEYLERİ İLE ATLETİK PERFORMANS ARASINDAKİ İLİŞKİNİN BELİRLENMESİ	Include		Original human research included an identifiable Turkish athlete population or separately extractable Turkish athlete subgroup, eligible inherited genetic or genomic data, and a sport-related phenotype.

Report ID	Title	Decision	Code	Reason for decision
Y0965	ELİT ERKEK TÜRK SPORCULARINDA ACTN3 R577X POLİMORFİZMİNİN DEĞERLENDİRİLMESİ	Include		Original human research included an identifiable Turkish athlete population or separately extractable Turkish athlete subgroup, eligible inherited genetic or genomic data, and a sport-related phenotype.
Y0970	Investigation of PPAR-Alpha gene polymorphism in female's soccer players.	Include		Original human research included an identifiable Turkish athlete population or separately extractable Turkish athlete subgroup, eligible inherited genetic or genomic data, and a sport-related phenotype.
Y0971	The Impact of Polymorphisms in AMPD1 and BMP2 Genes on Performance in Female Athletes.	Exclude	D8	The publication type was ineligible because it did not report original primary human data.
Y0981	Genetic differences in peroxisome proliferator-activated receptor alpha gene in endurance athletes (long distance runners) and power/endurance athletes (wrestlers, football players)	Include		Original human research included an identifiable Turkish athlete population or separately extractable Turkish athlete subgroup, eligible inherited genetic or genomic data, and a sport-related phenotype.
Y0984	HADO Sporcularında Alfa-Aktin-3 (ACTN3) rs1815739 Polimorfizminin Dağılımı	Include		Original human research included an identifiable Turkish athlete population or separately extractable Turkish athlete subgroup, eligible inherited genetic or genomic data, and a sport-related phenotype.
Y0992	Farklı Branşlardaki Sporcuların Kollajen Tip V gen (COL5A1) rs12722 Polimorfizmlerinin Dağılımı	Include		Original human research included an identifiable Turkish athlete population or separately extractable Turkish athlete subgroup, eligible inherited genetic or genomic data, and a sport-related phenotype.
Y1003	Türk Futbolcularda Alfa-Aktinin-3 (ACTN3) Ve Anjiyotensin Dönüştürücü Enzim (ACE) Polimorfizmleri Atletik Performans için Bir Biyobelirteç Olabilir mi?	Include		Original human research included an identifiable Turkish athlete population or separately extractable Turkish athlete subgroup, eligible inherited genetic or genomic data, and a sport-related phenotype.
Y1021	Determination of SLC6A4 promoter L/S polymorphism in professional volleyball players and comparison of the literature; Profesyonel voleybolcularda Slc6a4 promotor L/S polimorfizminin belirlenmesi ve literatur karsilasti.	Include		Original human research included an identifiable Turkish athlete population or separately extractable Turkish athlete subgroup, eligible inherited genetic or genomic data, and a sport-related phenotype.

Report ID	Title	Decision	Code	Reason for decision
Y1026	Mücadele sporcularında SLC6A4 geni 5-HTTLPR polimorfizmi ile saldırganlık düzeyleri arasındaki ilişkinin incelenmesi	Include		Original human research included an identifiable Turkish athlete population or separately extractable Turkish athlete subgroup, eligible inherited genetic or genomic data, and a sport-related phenotype.
Y1029	The evaluation of rs1805086 and rs1805065 polymorphisms in mstn gene and anthropometric properties of national and amateur arm wrestlers Evaluación de los polimorfismos rs1805086 y rs1805065 en el gen mstn y las propiedades antropométricas de los luchadores de brazo nacionales y amateurs	Include		Original human research included an identifiable Turkish athlete population or separately extractable Turkish athlete subgroup, eligible inherited genetic or genomic data, and a sport-related phenotype.
Y1032	THE EXAMINATION OF THE EFFECTS OF PPAR-A (RS4253778) ON SERUM _x000D_ LIPIDS IN ELITE CROSS-COUNTRY SKIERS	Include		Original human research included an identifiable Turkish athlete population or separately extractable Turkish athlete subgroup, eligible inherited genetic or genomic data, and a sport-related phenotype.
Y1037	Kas dokusu fizyolojisinde rol oynayan iki aday gen varyantının alt ekstremite kas enduransı yönünden farklı mevkilerde oynayan futbolcularda incelenmesi	Include		Original human research included an identifiable Turkish athlete population or separately extractable Turkish athlete subgroup, eligible inherited genetic or genomic data, and a sport-related phenotype.
Y1041	Türk Kökenli Futbolcularda Apolipoprotein-E Genotiplerinin Belirlenmesi	Include		Original human research included an identifiable Turkish athlete population or separately extractable Turkish athlete subgroup, eligible inherited genetic or genomic data, and a sport-related phenotype.
Y1064	Determination of HIF1-Ars11549465 Polymorphism in Elite_x000D_ Skiers	Include		Original human research included an identifiable Turkish athlete population or separately extractable Turkish athlete subgroup, eligible inherited genetic or genomic data, and a sport-related phenotype.
Y1086	Pathology-supported genetic testing presents opportunities for improved disability outcomes in multiple sclerosis	Exclude	D4	The study did not include an eligible athlete population.
Y1088	Association between NOS3 intron 4 VNTR variant and sports performance	Include		Original human research included an identifiable Turkish athlete population or separately extractable Turkish athlete subgroup, eligible inherited genetic or genomic data, and a sport-related phenotype.

Report ID	Title	Decision	Code	Reason for decision
Y1089	Preliminary Findings of the Distribution of Alpha-2-Adrenergic Receptor (ADRA2A) rs1800544 Polymorphisms in Kickboxing Players	Include		Original human research included an identifiable Turkish athlete population or separately extractable Turkish athlete subgroup, eligible inherited genetic or genomic data, and a sport-related phenotype.
Y1092	VEGFR2 Gene Polymorphism and Aerobic Performance in Turkish Elite Endurance Athletes	Include		Original human research included an identifiable Turkish athlete population or separately extractable Turkish athlete subgroup, eligible inherited genetic or genomic data, and a sport-related phenotype.
Y1097	Unravelling the interaction between the DRD2 and DRD4 genes, personality traits and concussion risk	Exclude	D2	No identifiable Turkish athlete population or separately extractable Turkish athlete subgroup was available.
Y0699	An integrated approach to assess the complications of deforming osteoarthritis in sports medicine; [Комплексный подход в оценке осложнений деформирующих остеоартрозов в спортивной медицине]	Pending		Full-text eligibility assessment was pending at the time of screening.
Y0701	The effect of CYP1A2 genotype on the ergogenic properties of caffeine during resistance exercise: a randomized, double-blind, placebo-controlled, crossover study	Pending		Full-text eligibility assessment was pending at the time of screening.
Y0705	Interleukin-6 rsl800795 polymorphism is not considered as a genetic biomarker in Turkish national skiing running athlete cohort	Pending		Full-text eligibility assessment was pending at the time of screening.
Y0707	Fetal Hemoglobin Modulators May Be Associated with Symptomology of Football Players with Sickle Cell Trait	Pending		Full-text eligibility assessment was pending at the time of screening.
Y0711	Apolipoprotein e Polymorphism and Oxidative Stress in Human Peripheral Blood Cells: Can Physical Activity Reactivate the Proteasome System through Epigenetic Mechanisms?	Pending		Full-text eligibility assessment was pending at the time of screening.
Y0712	INCREASED HEART RATE VARIABILITY IN OARSMEN IS ASSOCIATED TO UCP2 55VAL AND UCP3 -55T ALLELES; [ПОВЫШЕННАЯ ВАРИАБЕЛЬНОСТЬ СЕРДЕЧНОГО РИТМА У ГРЕБЦОВ АССОЦИИРОВАНА С АЛЛЕЛЯМИ UCP2 55VAL И UCP3 –55T]	Pending		Full-text eligibility assessment was pending at the time of screening.

Report ID	Title	Decision	Code	Reason for decision
Y0716	Frequency of gene ace i polymorphism i-d in athletes of different sports; [Frequência do polimorfismo i-d do gene eca i em atletas de diferentes esportes]; [Frecuencia del polimorfismo i-d del gen eca i en atletas de diferentes deportes]	Pending		Full-text eligibility assessment was pending at the time of screening.
Y0720	Muscle Work and Its Relationship with ACE and ACTN3 Polymorphisms Are Associated with the Improvement of Explosive Strength.	Pending		Full-text eligibility assessment was pending at the time of screening.
Y0725	Detection of epistasis between ACTN3 and SNAP-25 with an insight towards gymnastic aptitude identification	Pending		Full-text eligibility assessment was pending at the time of screening.
Y0727	Frequency of genetic polymorphism actn3 r577x and ace i/d in blind athletes of 5-a-side football; [Frecuencia del polimorfismo genético actn3 r577x y eca i/d en atletas ciegos de fútbol 5]	Pending		Full-text eligibility assessment was pending at the time of screening.
Y0729	Associations between the dopamine D4 receptor gene polymorphisms and personality traits in elite athletes.	Pending		Full-text eligibility assessment was pending at the time of screening.
Y0732	Frequency of occurrence of genetic polymorphisms associated with sports success in elite athletes in team sports; [Частота встречаемости генетических полиморфизмов, ассоциированных со спортивной успешностью, у спортсменов игровых видов спорта высших достижений]	Pending		Full-text eligibility assessment was pending at the time of screening.
Y0735	Genetic variants associated with exercise performance in both moderately trained and highly trained individuals	Pending		Full-text eligibility assessment was pending at the time of screening.
Y0738	Phenotypic convergence in Charcot-Marie-Tooth 2Y with novel VCP mutation	Pending		Full-text eligibility assessment was pending at the time of screening.
Y0743	Associations of Apolipoprotein E ε4 Genotype and Ball Heading With Verbal Memory in Amateur Soccer Players.	Pending		Full-text eligibility assessment was pending at the time of screening.
Y0744	Evaluation of ACTN3 R577X and ACE I/D polymorphisms in young Colombian athletes: An exploratory research.	Pending		Full-text eligibility assessment was pending at the time of screening.
Y0749	Common Myelin Regulatory Factor Gene Variants Predisposing to Excellence in Sports.	Pending		Full-text eligibility assessment was pending at the time of screening.

Report ID	Title	Decision	Code	Reason for decision
Y0750	Investigating the influence of mtDNA and nuclear encoded mitochondrial variants on high intensity interval training outcomes	Pending		Full-text eligibility assessment was pending at the time of screening.
Y0751	The Association of Aquaporin-1 Gene with Marathon Running Performance Level: a Confirmatory Study Conducted in Male Hispanic Marathon Runners	Pending		Full-text eligibility assessment was pending at the time of screening.
Y0754	Role of genetic polymorphisms of angiotensin converting enzyme to cardiovascular endurance in men ages 13-14 years after fartlek training for 3 months	Pending		Full-text eligibility assessment was pending at the time of screening.
Y0755	A Comparison of Training Modality and Total Genotype Scores to Enhance Sport-Specific Biomotor Abilities in Under 19 Male Soccer Players	Pending		Full-text eligibility assessment was pending at the time of screening.
Y0758	Can Genetic Testing Predict Talent? A Case Study of 5 Elite Athletes.	Pending		Full-text eligibility assessment was pending at the time of screening.
Y0766	Alpha-Actinin-3 Deficiency Might Affect Recovery from Non-Contact Muscle Injuries: Preliminary Findings in a Top-Level Soccer Team.	Pending		Full-text eligibility assessment was pending at the time of screening.
Y0770	Physical activity and amyotrophic lateral sclerosis: a Mendelian randomization study	Pending		Full-text eligibility assessment was pending at the time of screening.
Y0776	Genotype scores in energy and iron-metabolising genes are higher in elite endurance athletes than in nonathlete controls.	Pending		Full-text eligibility assessment was pending at the time of screening.
Y0777	The UKB envirome of depression: from interactions to synergistic effects	Pending		Full-text eligibility assessment was pending at the time of screening.
Y0783	Brain Perfusion Bridges Virtual-Reality Spatial Behavior to TPH2 Genotype for Head Acceleration Events	Pending		Full-text eligibility assessment was pending at the time of screening.
Y0784	Association between Polymorphism rs1799732 of DRD2 Dopamine Receptor Gene and Personality Traits among MMA Athletes.	Pending		Full-text eligibility assessment was pending at the time of screening.
Y0787	Analysis of drd2 gene polymorphism in the context of personality traits in a group of athletes	Pending		Full-text eligibility assessment was pending at the time of screening.

Report ID	Title	Decision	Code	Reason for decision
Y0788	Effect of COL5A1, GDF5, and PPARA Genes on a Movement Screen and Neuromuscular Performance in Adolescent Team Sport Athletes.	Pending		Full-text eligibility assessment was pending at the time of screening.
Y0790	Association of ACTN3 Polymorphism with Body Somatotype and Cardiorespiratory Fitness in Young Healthy Adults.	Pending		Full-text eligibility assessment was pending at the time of screening.
Y0791	Study of the association of gene polymorphism with the risk of non-communicable diseases in martial artists; [Изучение ассоциации полиморфизма генов с риском алиментарно-зависимых заболеваний у спортсменов-единоборцев]	Pending		Full-text eligibility assessment was pending at the time of screening.
Y0792	Recurrent ventricular tachycardia after catheter ablation in arrhythmogenic right ventricular cardiomyopathy: Scar progression or ineffective ablation?	Pending		Full-text eligibility assessment was pending at the time of screening.
Y0795	Whole genome sequencing of elite athletes	Pending		Full-text eligibility assessment was pending at the time of screening.
Y0799	Impact of hormonal contraceptives on urinary steroid profile in relation to serum hormone changes and CYP17A1 polymorphism	Pending		Full-text eligibility assessment was pending at the time of screening.
Y0802	Effects of CYP1A2 and ADORA2A Genotypes on the Ergogenic Response to Caffeine in Professional Handball Players.	Pending		Full-text eligibility assessment was pending at the time of screening.
Y0805	KCNA4 Gene Variant is Auxiliary in Endurance Running Performance Level	Pending		Full-text eligibility assessment was pending at the time of screening.
Y0806	A molecular insight in sport and athletic alpha – actin3 gene in students of Physical Education Faculty and bodybuilders	Pending		Full-text eligibility assessment was pending at the time of screening.
Y0807	A Potential Endurance Algorithm Prediction in the Field of Sports Performance.	Pending		Full-text eligibility assessment was pending at the time of screening.
Y0812	The Val66 and Met66 Alleles-Specific Expression of BDNF in Human Muscle and Their Metabolic Responsivity	Pending		Full-text eligibility assessment was pending at the time of screening.

Report ID	Title	Decision	Code	Reason for decision
Y0819	PPARD CC and ACTN3 RR genotype prevalence among elite soccer players	Pending		Full-text eligibility assessment was pending at the time of screening.
Y0821	Can a genetic profile be related to performance in young talent track and field athletes? A pilot study	Pending		Full-text eligibility assessment was pending at the time of screening.
Y0825	Is there a difference between power and endurance track and field runners according to their genetic predisposition in motivation and self-esteem?; [Esiste una differenza tra i corridori di atletica leggera e di resistenza sulla base della loro predisposizione genetica alla motivazione e nell'autostima?]	Pending		Full-text eligibility assessment was pending at the time of screening.
Y0828	Alpha-Actinin-3 R577X Polymorphism Influences Muscle Damage and Hormonal Responses After a Soccer Game	Pending		Full-text eligibility assessment was pending at the time of screening.
Y0829	Do ACE and CKMM gene variations have potent effects on physical performance in inactive male adolescents?	Pending		Full-text eligibility assessment was pending at the time of screening.
Y0833	Insulin-like growth factor axis genetic score and sports excellence	Pending		Full-text eligibility assessment was pending at the time of screening.
Y0834	Association of COA1 with Patellar Tendonitis: A Genome-wide Association Analysis	Pending		Full-text eligibility assessment was pending at the time of screening.
Y0837	ACTN3 R577X genotype and exercise phenotypes in recreational marathon runners	Pending		Full-text eligibility assessment was pending at the time of screening.
Y0838	Impact of the UGT2B17 polymorphism on the steroid profile. Results of a crossover clinical trial in athletes submitted to testosterone administration.	Pending		Full-text eligibility assessment was pending at the time of screening.
Y0841	Analysis of the football players' actual nutrition; [Анализ фактического питания спортсменов футбольной команды]	Pending		Full-text eligibility assessment was pending at the time of screening.
Y0844	Androgen receptor gene microsatellite polymorphism is associated with muscle mass and strength in bodybuilders and power athlete status.	Pending		Full-text eligibility assessment was pending at the time of screening.

Report ID	Title	Decision	Code	Reason for decision
Y0845	Team sport, power, and combat athletes are at high genetic risk for coronavirus disease-2019 severity	Pending		Full-text eligibility assessment was pending at the time of screening.
Y0848	Associations of uncoupling protein 2 ala55val and uncoupling protein 3-55C/T polymorphisms with heart rate variability in young oarsmen-a pilot study	Pending		Full-text eligibility assessment was pending at the time of screening.
Y0849	The correlation of blood pressure changes and cardiac morpho-functional restructuring in young athletes; [Связь между изменениями артериального давления и морфофункциональной перестройкой сердца у юных атлетов]	Pending		Full-text eligibility assessment was pending at the time of screening.
Y0852	Multidisciplinary prediction of running-related injuries using machine learning	Pending		Full-text eligibility assessment was pending at the time of screening.
Y0874	COMT and ACE (Epi)genetic Variation Is Associated with Cognitive and Metabolic Resilience in Swiss Tactical Athletes.	Pending		Full-text eligibility assessment was pending at the time of screening.
Y0879	Lifestyle behaviors and immune cell characteristics in type 2 diabetes risk: a Mendelian randomization study	Pending		Full-text eligibility assessment was pending at the time of screening.
Y0886	The Transforming Growth Factor β Genes and Susceptibility to Musculoskeletal Injuries in a Physically Active Caucasian Cohort.	Pending		Full-text eligibility assessment was pending at the time of screening.
Y0895	Genome-Wide Association Study of Vertical Jump Performance Among Elite Badminton Players	Pending		Full-text eligibility assessment was pending at the time of screening.
Y0896	Dopaminergic gene-epigenetic interactions and impulsivity traits in elite combat sport athletes.	Pending		Full-text eligibility assessment was pending at the time of screening.
Y0899	Approach to the Patient With Traumatic Brain Injury–induced Pituitary Dysfunction	Pending		Full-text eligibility assessment was pending at the time of screening.
Y0912	The influence of the Comt Val158Met Polymorphism on Heart rate variability Parameters, Psychoemotional Status, and Sports Burnout in Athletes	Pending		Full-text eligibility assessment was pending at the time of screening.

Report ID	Title	Decision	Code	Reason for decision
Y0939	ACE2 genetic polymorphisms and non-genetic factors were associated with a history of musculoskeletal injuries in high-performance athletes.	Pending		Full-text eligibility assessment was pending at the time of screening.
Y0951	ACTN3 ASSOCIATION ON MAXIMAL MUSCLE POWER, AFTER 6 WEEKS OF POWER TRAINING.	Pending		Full-text eligibility assessment was pending at the time of screening.
Y0952	Sox15 T/G may be a candidate gene polymorphism for athlete performance.	Pending		Full-text eligibility assessment was pending at the time of screening.
Y0953	Significance of ACTN3 gene and its relation with physiological and physical variables as a genetic marker for soccer players' selection at high altitudes.	Pending		Full-text eligibility assessment was pending at the time of screening.
Y0955	The "Warrior" COMT Val/Met Genotype Occurs in Greater Frequencies in Mixed Martial Arts Fighters Relative to Controls.	Pending		Full-text eligibility assessment was pending at the time of screening.
Y0959	ACE/ACTN3 GENETIC POLYMORPHISMS AND ATHLETIC PERFORMANCE OF FEMALE SOCCER PLAYERS'.	Pending		Full-text eligibility assessment was pending at the time of screening.
Y0962	Kadın Sporcularda AGTR2 Geni A/C Polimorfizminin Aerobik Dayanıklılık Performansı Üzerindeki Etkisi.	Pending		Full-text eligibility assessment was pending at the time of screening.
Y0964	ACTN3 R577X (rs1815739) Polymorphism and Athlete Status: An Additional Case–Control Association Study and Meta-Analysis.	Pending		Full-text eligibility assessment was pending at the time of screening.
Y0966	Análisis correlacional del perfil dermatoglífico y la potencia del equipo gimnasia artística femenino departamento del Tolima en Colombia.	Pending		Full-text eligibility assessment was pending at the time of screening.
Y0969	EVALUATING THE EFFECT OF THE CYP1A2 rs762551 GENE POLYMORPHISM ON ARROWHEAD AGILITY DRILL TEST PERFORMANCE IN PHYSICALLY ACTIVE YOUNG MEN.	Pending		Full-text eligibility assessment was pending at the time of screening.

Report ID	Title	Decision	Code	Reason for decision
Y0973	DETERMINATION OF THE GENETIC PROFILE OF MCT1 (SLC16A1) GENE A/T POLYMORPHISM AND GDF5 GENE T/C POLYMORPHISM RELATED TO CONNECTIVE TISSUE AND MUSCLE INJURY RISK IN YOUNG ATHLETES.	Pending		Full-text eligibility assessment was pending at the time of screening.
Y0975	ASSOCIATION BETWEEN MCT1 GENE POLYMORPHISM (rs1049434) WITH THE ATHLETIC PERFORMANCE OF ELITE TRACK AND FIELD ATHLETES.	Pending		Full-text eligibility assessment was pending at the time of screening.
Y0977	The Effect Of Heterozygosity For The Col1a1 Rs1107946 Polymorphism On Flexibility And Climber Status: 2521.	Pending		Full-text eligibility assessment was pending at the time of screening.
Y0980	The MCT1 gene Glu490Asp polymorphism (rs1049434) is associated with endurance athlete status, lower blood lactate accumulation and higher maximum oxygen uptake.	Pending		Full-text eligibility assessment was pending at the time of screening.
Y0983	Futbolcularda Anjiyotensinojen (AGT) rs699 Polimorfizm Dağılımının Belirlenmesi	Pending		Full-text eligibility assessment was pending at the time of screening.
Y0985	Elit Atletlerde SOD2 Rs4880 Polimorfizminin Yarışma Performansı ile İlişkisi	Pending		Full-text eligibility assessment was pending at the time of screening.
Y0993	Investigation of COL12A1 Gene Polymorphism Profile in Genetic Injury Susceptibility in Football Players Having Anterior Cruciate Ligament Surgery	Pending		Full-text eligibility assessment was pending at the time of screening.
Y0994	MITOCHONDRIAL HAPLOGROUP DISTRIBUTION IN TURKISH ELITE ATHLETES	Pending		Full-text eligibility assessment was pending at the time of screening.
Y0999	Genetics and athletic performance.	Pending		Full-text eligibility assessment was pending at the time of screening.
Y1000	Atletik performans ve spor genetiği	Pending		Full-text eligibility assessment was pending at the time of screening.
Y1001	Athletic performance, sports experience, and exercise addiction: An association study on ANKK1 gene polymorphism rs1800497	Pending		Full-text eligibility assessment was pending at the time of screening.

Report ID	Title	Decision	Code	Reason for decision
Y1002	Comparison of athletic performance of Turkish ice hockey players with ACE I/D (rs1799752), ACTN3 (rs1815739), PPARA (rs4253778) and HIF1A (rs11549465) polymorphisms	Pending		Full-text eligibility assessment was pending at the time of screening.
Y1004	Koşucularda ACTN3 ve ACE genlerinin sportif performansa etkisi	Pending		Full-text eligibility assessment was pending at the time of screening.
Y1006	Futbolcularda ACTN3 rs1815739 Polimorfizmi, Fiziksel Özellikler ve Mevki İlişkisinin Araştırılması: Bir Takım Örneklemi	Pending		Full-text eligibility assessment was pending at the time of screening.
Y1007	Evaluation of association between PPARGC1A gene polymorphism and competitive performance of elite athletes	Pending		Full-text eligibility assessment was pending at the time of screening.
Y1009	FUTBOLCULARDA SLC6A4 PROMOTÖR POLİMORFİZMİNİN DAĞILIMI	Pending		Full-text eligibility assessment was pending at the time of screening.
Y1010	Investigation of the effects of ACE gene polymorphism on athletic performance in active adult males	Pending		Full-text eligibility assessment was pending at the time of screening.
Y1012	Türk ironman triatlon sporcularında IL-6, HIF1A, MCT1, PPAR-a Polimorfizm dağılımının belirlenmesi	Pending		Full-text eligibility assessment was pending at the time of screening.
Y1013	Analysis of HIF1A (rs11549465) Polymorphism Distributions in Female Volleyball Players	Pending		Full-text eligibility assessment was pending at the time of screening.
Y1014	Kadın Futbolcularda Ppar-Alpha Geni Polimorfizminin İncelenmesi	Pending		Full-text eligibility assessment was pending at the time of screening.
Y1015	Sporda Genetik ve Darbeye Bağlı Olmayan Yumuşak Doku Yaralanmaları İlişkisi Üzerine 2026 Güncellemesi: GDF5 Gen Polimorfizm İncelemesi	Pending		Full-text eligibility assessment was pending at the time of screening.
Y1016	AKTİF YETİŞKİN ERKEKLERDE ACE GEN POLİMORFİZMİNİN ATLETİK PERFORMANS ÜZERİNDEKİ ETKİLERİNİN İNCELENMESİ	Pending		Full-text eligibility assessment was pending at the time of screening.
Y1017	Profesyonel Voleybolcularda Katekol-O-Metiltransferaz (COMT) rs4680 Polimorfizminin Belirlenmesi	Pending		Full-text eligibility assessment was pending at the time of screening.

Report ID	Title	Decision	Code	Reason for decision
Y1018	The relationship of polymorphism with explosive forces in ACTN3, ACE, and UCP3 genes in soccer players	Pending		Full-text eligibility assessment was pending at the time of screening.
Y1020	The Determination Of The Relationship Between Vo2max And The Angiotensin-Converting Enzyme Gene (ACE) rs1799752 Polymorphisms In The Turkish National Ice Hockey Sports Team	Pending		Full-text eligibility assessment was pending at the time of screening.
Y1022	EFFECTS OF GC RS2282679 GENE POLYMORPHISM ON YO-YO INTERMITTENT RECOVERY TEST 2 PERFORMANCE IN ACTIVE MEN	Pending		Full-text eligibility assessment was pending at the time of screening.
Y1024	NUTRIGENOMICS AND NUTRIGENETICS IN ATHLETE NUTRITION	Pending		Full-text eligibility assessment was pending at the time of screening.
Y1025	STRUCTURE OF GENES AND RELATION TO THE PHYSICAL ACTIVITY	Pending		Full-text eligibility assessment was pending at the time of screening.
Y1027	Sport nutrigenomics: personalized nutrition for athletic performance	Pending		Full-text eligibility assessment was pending at the time of screening.
Y1028	Distribution of dopamine receptor 2 DRD2 rs1800497 polymorphisms in professional football players	Pending		Full-text eligibility assessment was pending at the time of screening.
Y1031	The relationship between swimming styles and Il-6 Rs1800795 polymorphism in professional swimmers	Pending		Full-text eligibility assessment was pending at the time of screening.
Y1033	Investigation of Alpha-Actinin-3 (ACTN3) Gene Polymorphism in Bodybuilding Athletes: The Case of Samsun Province in Turkey	Pending		Full-text eligibility assessment was pending at the time of screening.
Y1038	Sports Nutrigenomic	Pending		Full-text eligibility assessment was pending at the time of screening.
Y1040	Advances in athlete genomics in 2019	Pending		Full-text eligibility assessment was pending at the time of screening.
Y1043	Collagen type 1 gene (COL1A1) rs1800012 polymorphism in long and short distance runners.	Pending		Full-text eligibility assessment was pending at the time of screening.

Report ID	Title	Decision	Code	Reason for decision
Y1045	Genetic factors and anterior cruciate ligament injury risk in professional football players: COL3A1 (rs1800255) and COL5A1 (rs12722) polymorphisms	Pending		Full-text eligibility assessment was pending at the time of screening.
Y1048	Sportif Performans ve BDNF İlişkisi.	Pending		Full-text eligibility assessment was pending at the time of screening.
Y1051	The GALNTL6 gene rs558129 polymorphism is associated with power performance	Pending		Full-text eligibility assessment was pending at the time of screening.
Y1053	GENETIC PROFILE OF HOCKEY PLAYERS AT DIFFERENT STAGES OF SPORT TRAINING	Pending		Full-text eligibility assessment was pending at the time of screening.
Y1054	Cellular aspects of muscle specialization demonstrate genotype–phenotype interaction effects in athletes	Pending		Full-text eligibility assessment was pending at the time of screening.
Y1055	ACE I/D and AGT rs699 Polymorphisms and Positional Associations in Handball	Pending		Full-text eligibility assessment was pending at the time of screening.
Y1056	Relationship Between Genes, Physical Activity & Metabolic Diseases	Pending		Full-text eligibility assessment was pending at the time of screening.
Y1058	The Determination of IL-6 rs1800795 Polymorphism Distribution in Turkish National Cross-Country Skiing Athletes Sub-groups Created Referring to the 1km CCSTAs	Pending		Full-text eligibility assessment was pending at the time of screening.
Y1059	Determination of the Anxiety–Related SLC6A4 Gene Promoter “S” and “L” Alleles in Football Players	Pending		Full-text eligibility assessment was pending at the time of screening.
Y1061	Circadian clock gene variants may affect circadian fluctuations of anaerobic performance	Pending		Full-text eligibility assessment was pending at the time of screening.
Y1065	Genetic and Physiological Predictors of Bioenergetic Adaptation Skeletal Muscles in Athletes of Cyclic Sports	Pending		Full-text eligibility assessment was pending at the time of screening.
Y1069	Evaluation of the effects of emotional and violence-related genes in athletes	Pending		Full-text eligibility assessment was pending at the time of screening.
Y1070	Candidate genes and single-nucleotide gene variants associated with muscle and tendon injuries in cyclic sports athletes	Pending		Full-text eligibility assessment was pending at the time of screening.

Report ID	Title	Decision	Code	Reason for decision
Y1071	The distribution of brain-derived neurotrophic factor rs6265 polymorphism in Turkish Volleyball players	Pending		Full-text eligibility assessment was pending at the time of screening.
Y1072	Genetics and athleticism: Exploring the role of DNA in sports talent and training adaptation	Pending		Full-text eligibility assessment was pending at the time of screening.
Y1073	Candidate genes associated with athletes' skeletal muscle functions regulation	Pending		Full-text eligibility assessment was pending at the time of screening.
Y1077	Association of alpha-actinin-3 genotype with muscle mass and physical function in community-dwelling older adults	Pending		Full-text eligibility assessment was pending at the time of screening.
Y1078	Genetic susceptibility of MMP3 (rs3025058) variant allele in male football players with multiple ACL surgeries: a PCR-RFLP analysis	Pending		Full-text eligibility assessment was pending at the time of screening.
Y1082	The speed-gene study: methods, study design and preliminary results	Pending		Full-text eligibility assessment was pending at the time of screening.
