# Supplementary material for: Sports genomics research in Turkish athlete populations 2019 to 2026: a systematic review
Source: Front Genet. 2026 Oct 23;17:1921682. doi: 10.3389/fgene.2026.1921682 (PMC13739473; doi:10.3389/fgene.2026.1921682)
Supplement: Supplementary file 4 [file DataSheet4.pdf]

Supplementary Table S4 Cohort Linkage and Population Totals

Six confirmed shared-cohort clusters encompassed 18 reports. Cohort linkage was used to avoid double counting in population-level totals; reports were retained when they provided non-overlapping eligible genetic or phenotypic information.

Population totals after confirmed cohort merging: 58 operationally unique study populations; 3,201 athletes; 3,683 non-athlete controls; 6,884 total participants.

Population ID	Linked reports	Unique population	Evidence for linkage
POP-01	Y0127; Y0310	67 elite Turkish male wrestlers	Same n=67 male wrestlers and Gazi ethics protocol 2023/642.
POP-02	Y0063; Y0284; Y0359; Y0464; Y0486; Y1007; Y0975; Y1001	60 elite Turkish track and field athletes + 20 controls	Same Turkish Athletics Federation sample: 31 sprint/power athletes + 29 endurance athletes and 20 controls.
POP-04	Y0965; Y0981	158 elit erkek sporcu + 56 kontrol	Same 53 wrestlers, 71 football players, and 34 runners; ethics protocol 217 (23 May 2012).
POP-12	Y1032; Y1064	34 national cross-country skiers	Same national-team training camp, 23 men and 11 women; cohort-level confirmation.
POP-16	Y0016; Y0468	50 professional female volleyball players + 557 controls	Same five clubs, same 557 Turkish Genome Project controls, and ethics protocol 2025-110.
POP-10	Y1026; MAN-04	40 elite combat-sport athletes	Same SLC6A4 5-HTTLPR/aggression question, same n=40, and same Ondokuz Mayıs research setting; thesis-article report pair.

Note. Potential but unconfirmed overlaps were not merged. The report-to-population assignment for all 70 reports is retained in Supplementary Data S1.
